# Identification of Genes That Promote or Antagonize Somatic Homolog Pairing Using a High-Throughput FISH–Based Screen

**DOI:** 10.1371/journal.pgen.1002667

**Published:** 2012-05-10

**Authors:** Eric F. Joyce, Benjamin R. Williams, Tiao Xie, C.-ting Wu

**Affiliations:** 1 Department of Genetics, Harvard Medical School, Boston, Massachusetts, United States of America; 2 Department of Systems Biology, Harvard Medical School, Boston, Massachusetts, United States of America; 3 Image and Data Analysis Core, Harvard Medical School, Boston, Massachusetts, United States of America; Stowers Institute for Medical Research, United States of America

## Abstract

The pairing of homologous chromosomes is a fundamental feature of the meiotic cell. In addition, a number of species exhibit homolog pairing in nonmeiotic, somatic cells as well, with evidence for its impact on both gene regulation and double-strand break (DSB) repair. An extreme example of somatic pairing can be observed in *Drosophila melanogaster*, where homologous chromosomes remain aligned throughout most of development. However, our understanding of the mechanism of somatic homolog pairing remains unclear, as only a few genes have been implicated in this process. In this study, we introduce a novel high-throughput fluorescent in situ hybridization (FISH) technology that enabled us to conduct a genome-wide RNAi screen for factors involved in the robust somatic pairing observed in Drosophila. We identified both candidate “pairing promoting genes” and candidate “anti-pairing genes,” providing evidence that pairing is a dynamic process that can be both enhanced and antagonized. Many of the genes found to be important for promoting pairing are highly enriched for functions associated with mitotic cell division, suggesting a genetic framework for a long-standing link between chromosome dynamics during mitosis and nuclear organization during interphase. In contrast, several of the candidate anti-pairing genes have known interphase functions associated with S-phase progression, DNA replication, and chromatin compaction, including several components of the condensin II complex. In combination with a variety of secondary assays, these results provide insights into the mechanism and dynamics of somatic pairing.

## Author Summary

In addition to their number and structure, the position and spatial dynamics of chromosomes are under tight control, as direct interactions between chromosomes can contribute to the activation or repression of genes. Here, we focus on a particular type of interaction, known as somatic homolog pairing, which occurs between the maternal and paternal copies of chromosomes. While the role of somatic pairing on downstream homology-driven processes is well-established, there is much to be learned about how homologous chromosome segments find each other, physically align, and form stable pairing interactions within somatic cells. Taking advantage of a novel high-throughput FISH technology and the fact that homologous chromosomes are intimately paired along their lengths in the somatic cells of Drosophila, we have conducted a screen for factors that are important for the fidelity of somatic pairing. Ultimately, the characterization of these pairing genes will shed light on the mechanism of pairing, as well as pairing-mediated processes that have implications for development and disease. Finally, the efficacy of our screen for pairing genes suggests that the high-throughput FISH technology described here will prove useful for studying forms of nuclear organization and chromosome positioning beyond pairing.

## Introduction

Pairing of homologous chromosomes is a fundamental aspect of nuclear organization. Although most well-known for its role in chromosome segregation during meiosis, studies have also documented homolog pairing in somatic tissues [1]–[5]. The most dramatic examples can be observed in Dipteran insects, such as *Drosophila melanogaster*, where homologous chromosomes are intimately paired in virtually all cell types throughout development [4], [6], [7]. Importantly, these pairing interactions have been shown to affect gene regulation at a number of loci through a process termed transvection [1], [2], [8]–[13] and influence the repair of somatic DNA double-strand breaks [14].

In contrast to Drosophila, the homologous pairing of any particular chromosome or chromosomal region in most organisms, if it occurs at all, is transient and localized. For example, short-lived homolog associations have been implicated in both mammalian X-inactivation 15–19 and immunoglobulin gene recombination during B cell development [20]. Additionally, there is evidence that mammalian chromosomes of somatic cells can colocalize, perhaps even undergo homologous pairing, at specific stages of the cell cycle [21]–[25], consistent with observations indicating that the mammalian nucleus can arrange its chromosomes nonrandomly [26]–[28]. One possible explanation for the relatively modest level of pairing in mammals as compared to that found in Drosophila is that mammalian cells support mechanisms that inhibit interchromosomal interactions throughout most of development [29], [30]. Indeed, identification of the condensin II subunit, Cap-H2, as a protein in Drosophila that antagonizes polytene chromosome alignment and transvection supports the idea that homologous interactions can be actively inhibited [31], perhaps even in a cell-cycle regulated fashion [32]. What remains unclear is why and how pairing is generally prohibited in most organisms and yet is so robust and genome-wide in Drosophila.

One strategy to better understand the mechanistic and functional basis of somatic pairing and its downstream role in transcriptional regulation is to identify the genes involved. To date, only two proteins have been directly implicated in promoting somatic pairing in Drosophila: Suppressor of Hairy Wing (Su(Hw)) [33] and Topoisomerase II (Top2) [30]. Using fluorescent in situ hybridization (FISH) targeting euchromatic loci in order to provide a direct measure of somatic pairing, loss of Su(Hw) and inhibition of Top2 have both been shown to partially compromise homolog pairing in tissues and cell culture, respectively. Intriguingly, Top2 has been suggested to modulate the activity of Su(Hw) [34], indicating that these two proteins may function together. Aside from these findings, FISH-based searches for pairing factors, one via a candidate gene approach [35] and a second entailing a whole-genome screen in early embryos [36], have failed to identify genes whose products control somatic pairing.

Searches for genes involved in somatic pairing have also taken advantage of transvection-associated phenotypes and, while not a direct measure of pairing, these phenotypes have enabled genetic studies to isolate additional candidates. These include genes encoding proteins that mediate long-range interactions, such as Zeste and the Polycomb group proteins [2], , although direct involvement of such candidates in homolog pairing has yet to be obtained. What has been observed are correlations between the cell cycle and relative levels of somatic pairing [2], [4], [32], [43]–[46]. For example, high levels of somatic pairing may require a long interphase or an uninterrupted period during which chromosomes are still decondensed. Pairing may even be disrupted during S-phase and mitosis, lending further support that pairing is possibly regulated through the cell cycle, although direct genetic evidence for such a link is lacking. Cohesin is a protein complex that has also been implicated in long-range interactions as well as the tethering of sister chromatids in both mitosis and meiosis [47], [48], and, perhaps most suggestively, the mechanism and control of meiotic homolog pairing [49]–[52]. Nevertheless, there is no direct evidence for the involvement of the cohesin complex in somatic pairing, and it remains unclear if meiotic pairing and somatic pairing are mechanistically similar [4].

Here, we present a genome-wide FISH-based screen in Drosophila cell culture to identify the factors involved in the somatic pairing of heterochromatic regions. This screen was made possible through the development of a high-throughput FISH technology that permits chromosomal positions to be directly visualized in a 384-well format. Combined with RNAi, this approach permitted us to screen two heterochromatic regions simultaneously for double-stranded RNAs (dsRNAs) that alter the fidelity and/or strength of somatic homolog pairing. Using an increased number of FISH signals per nucleus as a readout for decreased pairing, we report the identification of 40 candidate ‘pairing promoting genes,’ none of which had been previously associated with pairing functions. Importantly, many of these genes were also found to influence pairing at euchromatic regions, revealing a potentially strong mechanistic overlap between heterochromatic and euchromatic pairing. In addition, we identified 65 candidate ‘anti-pairing genes,’ which when knocked down enhance pairing, consistent with a wild-type function of antagonizing somatic pairing interactions. We propose a model in which interchromosomal associations are mediated by a dynamic interplay between groups of proteins with opposing functions: those that induce or augment pairing and others whose normal function is to disrupt pairing. This perspective suggests that the difference between Drosophila and other organisms may be a shift in the balance of gene function. Finally, in combination with a variety of secondary assays, the identification of these proteins has provided insights into the mechanism and dynamics of somatic pairing, pointing to an intriguing connection between the progression of the cell cycle and the control of somatic pairing.

## Results

### Heterochromatic pairing in Drosophila cell culture

The design of our studies began with an earlier observation that the onset of pairing in the Drosophila embryo does not require the zygotic expression of any particular gene but relies instead on parental contributions [36]. This finding suggested that traditional genetic screens for genes involved in pairing may not be straightforward, arguing for a cell culture- and FISH-based alternative. For this study, we chose the Drosophila Kc_167_ cell line due to its amenability to RNAi [53] and capacity to support high levels of pairing, despite being predominantly tetraploid [30]. In fact, it was in this cell line that our previous study identified Top2 as a gene important for somatic pairing [30].

**Figure 1:**
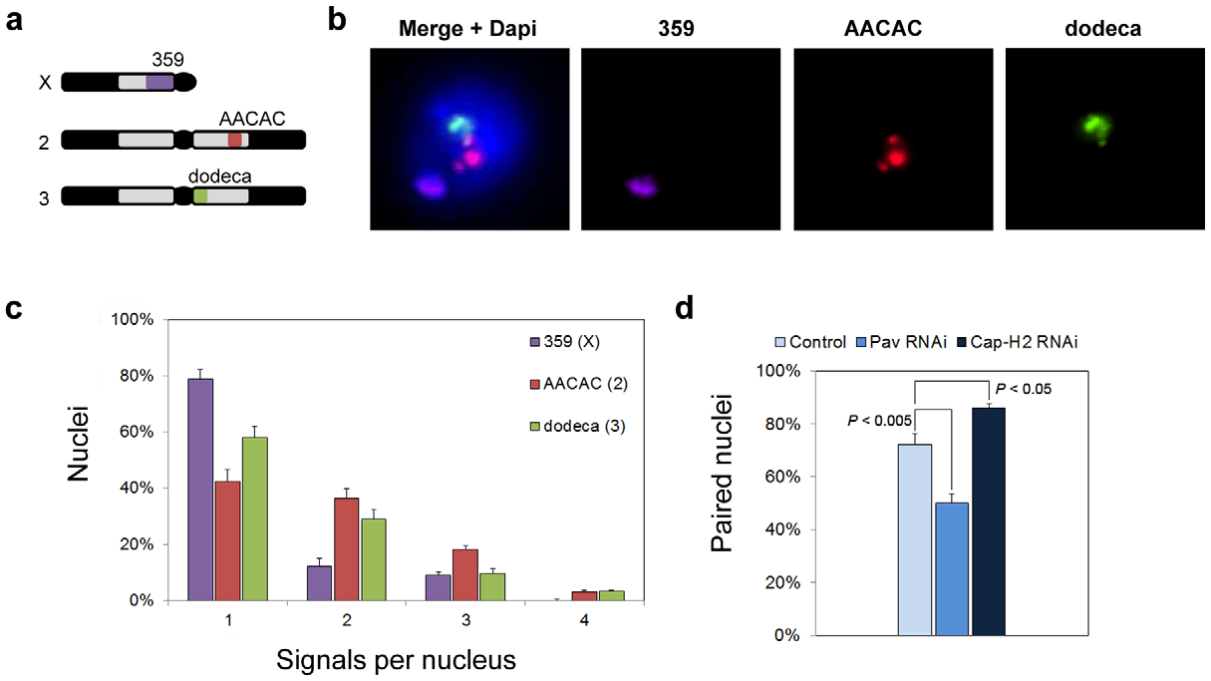
Heterochromatic pairing in Drosophila cell culture. a, Drosophila karyotype (Y and 4th chromosome not shown) and targets of heterochromatic probes. b, Flattened image of Kc_167_ nucleus stained with DAPI and FISH targeting 359, AACAC, and dodeca. c, Percentage of Kc_167_ nuclei ± standard deviation (SD) with the indicated number of signals per nucleus representing 359, AACAC, and dodeca. d, dsRNA directed against *pav* reduces the percentage of paired nuclei (one signal or two signals ≤1 µm apart) at dodeca in Kc_167_ cells (*P*<0.005), whereas dsRNA directed against *cap-H2* increases the percentage of paired nuclei (*P*<0.05). The data are from three trials.

Our analysis also required chromosomal targets that would produce robust, reproducible FISH signals. For this reason, we chose sequences of the centromeric heterochromatin, which make ideal FISH targets due to their great abundance, simplicity, and chromosome specificity [54]. We designed FISH probes, 15 to 35 bases in length, against three heterochromatic sequences: the 359, AACAC, and dodeca repeats of the X, 2^nd^, and 3^rd^ chromosomes, respectively (Materials and Methods; Figure 1). Using these probes, 80, 42, and 58% of Kc_167_ nuclei gave a single FISH signal at 359, AACAC, and dodeca, respectively, indicating close homolog alignment (Figure 1). We note that these levels of pairing are greater than those previously observed for heterochromatic sequences in Kc_167_ cells [30], possibly owing to the high specificity of our probes. However, consistent with this previous study, these levels of heterochromatic pairing are below those typically found at euchromatic regions (with the exception of pairing at 359), raising the possibility that heterochromatic regions may pair less often, pair more slowly, and/or even rely on mechanisms that differ from those responsible for pairing at euchromatic regions [30]. We reasoned that targeting these regions with FISH should allow us to detect either a reduction or an increase in pairing, thus identifying heterochromatic and, possibly, euchromatic pairing factors as well.

### Identification of Pav and Cap-H2 as putative regulators of heterochromatic pairing

We next conducted a pilot screen using dsRNA to knock down transcript levels of genes that had been previously shown to be important for the pairing of euchromatic regions. For example, RNAi inhibition of either Top2 [30] or the kinesin-like protein Pavarotti (Pav) (Williams, BR, unpublished) had been found to reduce euchromatic pairing levels in cell culture, whereas RNAi inhibition of Cap-H2 had been shown to antagonize euchromatic pairing *in vivo* [31]. Cells were incubated with dsRNA for 4 days, an extent of time known to reduce protein levels by >80% [55], [56], after which they were fixed, subjected to FISH targeting the dodeca satellite, and then scored by visual examination. Nuclei were considered paired when they contained only a single FISH signal or when the center-to-center distance between all pairs of FISH signals was ≤1.0 µm, a threshold selected based on control nuclei (72% paired). From these analyses, Pav was shown to be important for heterochromatic pairing, as indicated by a decrease in the number of paired nuclei to 50% following depletion by RNAi (*P*<0.005; Figure 1), while Cap-H2 was shown to antagonize heterochromatic pairing, as revealed by an increase in the number of paired nuclei to 86% (*P*<0.05; Figure 1), consistent with the role of condensin II *in vivo* [31]. Interestingly, we found that RNAi depletion of Top2 had no effect on the pairing frequencies observed at the heterochromatic regions of the X, 2nd, or 3rd chromosomes (data not shown) even though it reduced pairing at the euchromatic 28B region from 85% to 58% (*P*<0.005), confirming efficient knockdown of Top2.

### High-throughput FISH screen to identify genes involved in heterochromatic pairing

**Figure 2:**
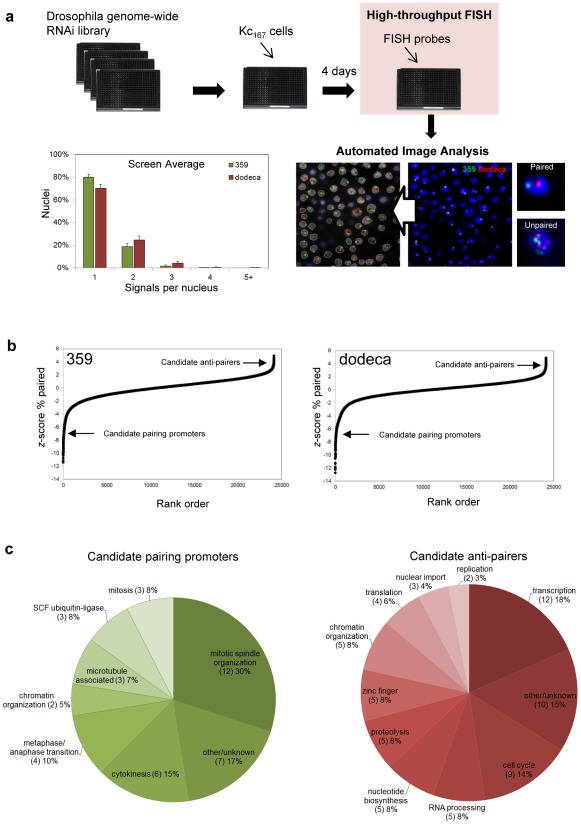
RNAi–mediated FISH–based screen of Drosophila cells for heterochromatic pairing factors. a, Experimental design with representative image before and after automated identification of nuclei and FISH signals. The screen averages for signals per nucleus obtained with probes targeting 359 and dodeca. b, Rank-order plot of each dsRNA in the primary screen, where negative *z*-scores indicate a reduction in paired nuclei (corresponding to candidate pairing promoters) and positive *z*-scores indicate an increase in paired nuclei (corresponding to candidate anti-pairers). c, Functional classifications of the candidate pairing promoters and anti-pairers.

The identification of Pav and Cap-H2 in our pilot run argued that a whole-genome screen in Kc_167_ cells should reveal genes involved in the somatic pairing of Drosophila heterochromatic regions. To this end, we developed a high-throughput FISH technology, allowing FISH assays to be performed in a 384-well format using a protocol that can be carried out, from fixation to imaging, within five hours. This strategy also enabled us to target two different heterochromatic regions simultaneously using probes against the 359 and dodeca repeated elements (Materials and Methods). We applied this technique to plates seeded with the well-characterized dsRNA whole-genome library of the Harvard Drosophila RNAi Screening Center (Figure 2), which represents 13,912 genes, at an average of 1.7 dsRNAs per gene, in a total of 66 plates. The screen was conducted in duplicate, each plate including dsRNAs against *pav* and *cap-H2* as positive controls for increased and decreased FISH signals, respectively. Wells containing dsRNAs against GFP, *lacZ*, and no dsRNA were used as negative controls, with no difference in pairing levels observed amongst them, suggesting that dsRNA in itself does not affect pairing (Table S1). A total of 50,668 FISH assays were conducted in the primary screen.

To automate scoring, we generated a custom MATLAB program to identify and count the number of FISH signals per nucleus (Materials and Methods), with an average of 928±185 nuclei being imaged per dsRNA. Computer algorithms were then used to calculate several parameters of pairing, such as the percentage of nuclei containing one, two, three, four, and ≥ five FISH signals, in order to detect different patterns of pairing as well as degrees of unpairing. Because we expected the unpairing of homologs to increase the distance between signals as well, we also incorporated a parameter that calculates the pairwise distances between multiple signals. Finally, as aneuploidy may affect the number of FISH signals per nucleus, the size of DAPI signals was recorded to monitor both increases and decreases in nuclear volume.

The screen average for the percentage of nuclei exhibiting a single FISH signal was 80±3% and 70±3% at 359 and dodeca, respectively, with the low variability demonstrating the reproducibility of the automated FISH counts (Figure 2). As the genes involved in somatic pairing are not expected to be clustered in any particular plate, we used the variation within each plate to assign a *z*-score (number of standard deviations by which the result differs from the mean value for the entire plate) to each well. Rank-order analysis of the primary genome-wide screening results demonstrated that the majority of dsRNAs had no effect on the number of FISH signals per nucleus, suggesting that pairing is not commonly disrupted by RNAi (Figure 2). However, we identified 372 dsRNAs that resulted in a significantly decreased number of nuclei with a single FISH signal (*z-*score ≥2.0), as would be expected for those that target genes important for somatic pairing. A second group of 63 dsRNAs resulted in a significantly increased number of single-signal nuclei (*z*-score ≤−2.0), as expected for those that target genes required for suppressing somatic pairing levels (Table S2). Together, the 435 dsRNAs that affected pairing targeted 352 annotated genes in the Drosophila genome.

### Validation and functional classification of candidate pairing factors

Because processed dsRNAs can produce off-target effects by cross-hybridizing with sequences corresponding to more than one gene [57], the 352 candidate genes identified in our primary screen were targeted with 1–2 additional non-overlapping dsRNAs. The validations were conducted in triplicate in 384-well plates with FISH probes targeting both the 359 and dodeca loci, and only those dsRNAs producing a significant increase or decrease (*P*≤0.05) of the percentage of single-signal nuclei compared to untreated control wells were considered “hits” (Materials and Methods for specific cut-off criteria). This narrowed our focus to 105 genes: 40 genes identified as candidate promoters of pairing, or ‘pairing promoters’, and 65 genes identified as candidate suppressors of pairing, or ‘anti-pairers’ (Table S3, Table S4). These data suggest that less than 1% of the Drosophila genome is directly or indirectly involved in somatic pairing of heterochromatic regions.

RNAi disruption of only 16% of the candidate pairing promoters significantly affected pairing at the 359-bp repeat on the X chromosome, estimated to be ∼11 Mb in length. Disruption of 98% of the candidates, however, were found to affect pairing at the dodeca locus, of unknown size, suggesting dodeca may represent a sensitized region that is more likely to unpair. Indeed, control pairing levels were significantly lower at dodeca as compared to those at 359 (Figure 1). Moreover, a positive correlation was found between the strongest pairing hits for dodeca and those that affected 359 (Figure S1).

Further examination of the 40 candidate pairing promoters revealed that 28 (70%) encode proteins with known or expected roles in cell division (Figure 2), the large majority of which are involved in mitotic spindle organization (12), cytokinesis (6) and the metaphase/anaphase transition (4). Of the remaining 12 (30%), 8 are known components of other cellular processes, including 3 subunits of the SCF ubiquitin-ligase complex. As for the 65 candidate anti-pairers, whose knockdown resulted in a decreased number of FISH signals, we hypothesize that they have wild-type functions that antagonize somatic pairing interactions. The most striking enrichments were for gene functions linked to S-phase progression (16, 26%), including cell cycle factors necessary for the G1/S transition (9), nucleotide biosynthesis (5), and replication (2) (Figure 2). Genes associated with transcription (12, 18%) or transcript processing (5, 8%) were also particularly prominent, with a further 5 (8%) genes encoding zinc finger proteins with potential roles in transcription (Figure 2). Some of these proteins could be required for sustained expression of the S-phase regulators. Of the remaining 32 genes, 5 (8%) are associated with proteolysis, 3 (5%) are involved in nuclear import, and 5 (8%) have roles in chromatin organization, including the condensin II subunits Cap-H2 and Cap-D3 and core condensin subunit SMC2.

These data suggest that somatic pairing of heterochromatic regions requires a complex network of genes that can promote as well as antagonize interchromosomal interactions. Below we describe our candidate pairing promoting and anti-pairing genes, and address their relationships to both heterochromatic and euchromatic pairing, aneuploidy, heterochromatin clustering, cell cycle progression, and each other. Taken together, these candidates point to an intriguing connection between the progression of the cell cycle and the control of somatic pairing as well as reveal an extensive overlap between heterochromatic pairing factors with those important for pairing at euchromatic regions.

### Candidate genes important for somatic pairing

#### SCF ubiquitin-ligase

**Figure 3:**
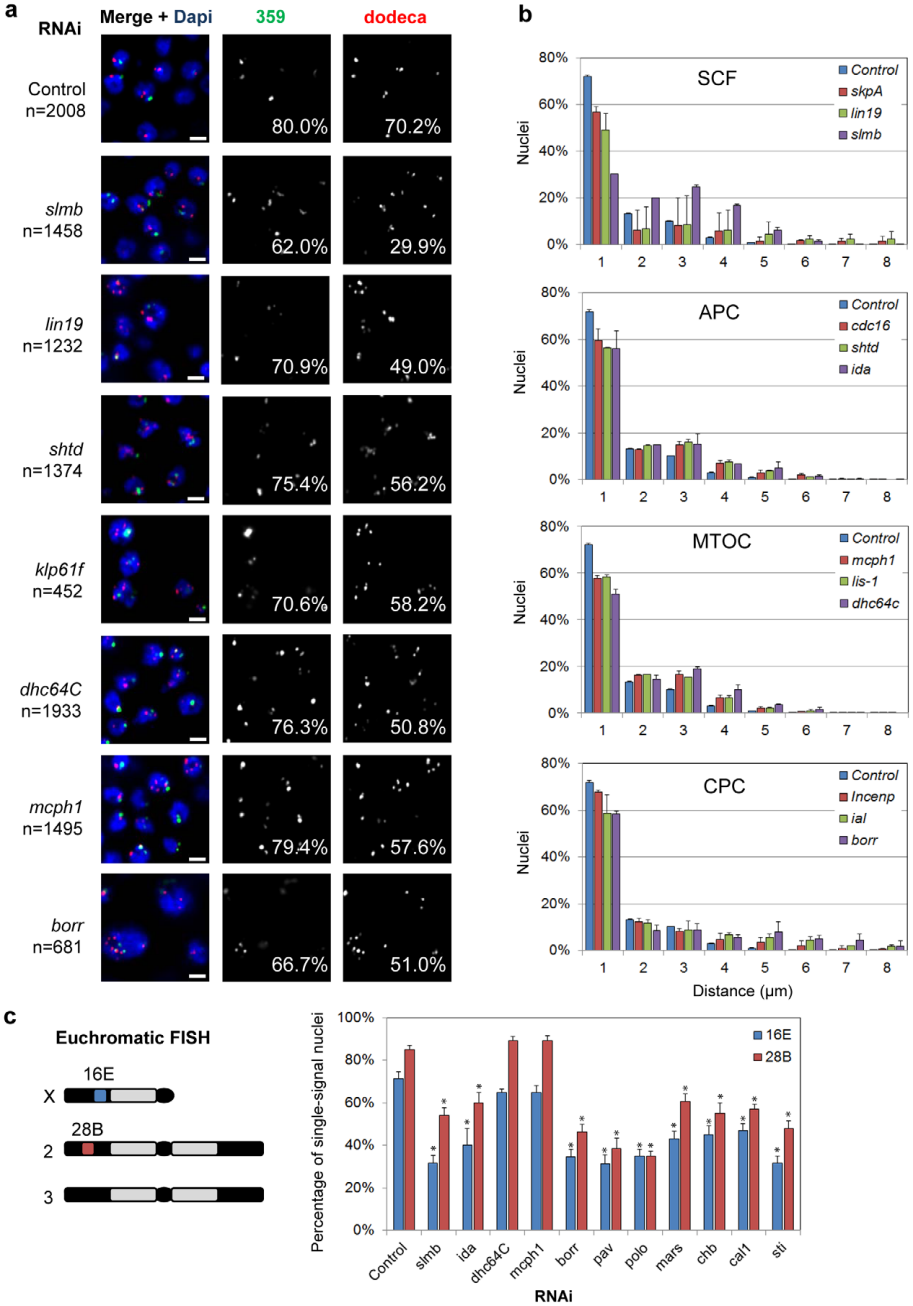
RNAi of candidate pairing promoters disrupts pairing. a, Representative FISH images are shown for RNAi knockdown of candidate pairing promoters (*slmb*, *lin19*, *shtd*, *klp61f*, *dhc64C*, *mcph1*, *borr*), where the number of FISH signals per nucleus is increased compared to control. The percentage of single-signal nuclei is noted for both 359 and dodeca. n denotes number of nuclei scored. Scale bars equal 5 µm. b, Relative frequencies of interhomolog distances (unpaired = two signals >1.0 µm apart) based on dodeca FISH ± SD for three tests. dsRNA targets are either grouped based on known interactions (SCF, APC, CPC) or localization patterns of the proteins they encode (MTOC). All significantly reduced the percentage of paired nuclei compared to control (*P*<0.05). c, Chromosomal targets of euchromatic FISH probes 16E and 28B and graph displaying the percentage of single-signal nuclei ± SD following RNAi. Asterisks denote a significant reduction from control (*P*<0.05). A minimum number of 100 nuclei were scored for each dsRNA.

dsRNAs targeting *slmb* and *lin19* were the two strongest hits identifying candidate pairing promoters (Table S3). We also identified Drosophila SKPA (Table S3), which physically interacts with SLMB and LIN19 [58]. Together, these candidates represent three of the four proposed components of the Drosophila SCF E3 ubiquitin-ligase complex, which targets signaling molecules and cell cycle regulators for degradation [58], [59]. We found that RNAi knockdown of *slmb*, *lin19*, and *skpA* reduced the percentage of nuclei with a single dodeca signal from 70% to 30% (*P* = 0.0001), 49% (*P* = 0.0075), and 56% (*P* = 0.002), respectively (Figure 3). Similar changes were also observed with probes targeting the 359 repeat (Figure 3, Table S3).

We reasoned unpairing of homologs would also increase the distance between FISH signals in addition to increasing their number per nucleus. Of those nuclei with multiple dodeca FISH signals in SLMB, LIN19, and SKPA-depleted cells, 99% exhibited two signals >1 µm apart and were therefore considered unpaired. In fact, 25% of SLMB-depleted cells had two signals >4 µm apart, a distance rarely observed in control nuclei (4%, *P*<0.0001; Figure 3). We further found that, despite an increase in the number of FISH signals, SLMB-depleted cells also exhibited reduced nuclear volumes (587±256 µm^3^) compared to that of controls (886±439 µm^3^, *P*<0.0001). Thus, these data point to a novel role for the SCF complex in controlling the organization and structure of interphase nuclei.

Finally, to investigate the contribution of the SCF complex to euchromatic pairing, we conducted FISH targeting two euchromatic regions, 16E and 28B, on the X and 2^nd^ chromosome, respectively, following *slmb* RNAi. Pairing was perturbed at both loci as observed by a reduction in the percentage of single-signal nuclei from 71 to 32% at 16E and from 85 to 54% at 28B (*P*<0.05 each; Figure 3), indicating the SCF complex is important for homologous pairing of both heterochromatic and euchromatic regions.

#### Anaphase-Promoting Complex

The Anaphase-Promoting Complex (APC) contains 11–13 proteins that, similar to SCF, function together as an E3 ubiquitin ligase, which targets cell cycle proteins for degradation. Our validation screen identified three components of the APC as candidate pairing promoters; RNAi depletion of SHTD (Drosophila APC1), CDC16, and IDA (Drosophila APC5) each reduced the percentage of single-signal nuclei (56–59%, *P*<0.04) and paired nuclei (all signals ≤1 µm apart) at dodeca (Figure 3, Table S3). A reduction in pairing, while not significant, was also observed with FISH targeting 359 (Figure 3, Table S3). Euchromatic pairing was also disrupted following IDA depletion, reducing the percentage of single-signal nuclei from 71 to 40% at 16E and from 85 to 60% at 28B (*P*<0.05), suggesting that, similar to SCF, proper APC function is important for pairing at both heterochromatic and euchromatic regions (Figure 3).

Although not confirmed in our validation screen, four additional members of the APC (FZY, APC10, APC4, and CDC27) were identified in our primary screen, with depletion of each causing a significant increase in the number of FISH signals per nucleus for both 359 and dodeca (Table S2), further supporting the role of APC in heterochromatic pairing. Our failure to identify these subunits in our validation screen is most likely due to the high stringency of the cut-off or inefficiency of the dsRNAs used in the validation studies. As seen below, different variants of the APC complex may have different and even opposing roles in pairing, which may further complicate the RNAi phenotypes of individual subunits.

The APC targets a different substrate spectrum for degradation in mitosis and interphase through interactions between the core APC subunit CDC27 and one of two adaptor proteins, CDC20 (mitotic) and CDH1 (interphase) [60]–[62]. We were intrigued to find that depletion of Drosophila CDC20 (FZY) reduced the percentage of paired nuclei at dodeca to 57% (*P* = 0.0002) (Table S2), whereas knockdown of Drosophila CDH1 (RAP) had the opposite effect, increasing pairing levels to 81% (*P* = 0.0097) (Table S4).

#### Proteins required for microtubule organization and chromosome segregation

The organization of microtubules has well-established roles in chromosome alignment and organization in both interphase and mitotic cells. We identified three genes that encode proteins associated with the microtubule organizing center (MTOC), which is required for proper microtubule nucleation and establishment of a bipolar spindle: *mcph1*, the Dynein motor protein encoding gene *dhc64C*, and the Dynein regulator *Lis-1*. Knockdown of each of these genes reduced the percentage of single-signal and paired nuclei at dodeca to 51–58% (*P*<0.004), while not significantly affecting the number of signals at 359 (Figure 3, Table S3). Significant increases in the distances between dodeca FISH signals were also found in MCPH1, Dhc64C, and LIS-1-depleted cells (*P*<0.02; Figure 3). Interestingly, however, euchromatic pairing frequencies at 16E and 28B were not affected by either *mcph1* or *dhc64C* RNAi (Figure 3), suggesting these genes may function specifically in pairing between heterochromatic regions.

Our screen also identified proteins belonging to the well-described chromosomal passenger complex (CPC), which is involved in various aspects of mitosis, including chromosome alignment, spindle assembly, and the completion of cytokinesis [63]. Specifically, we found that dsRNAs targeting *ial* (Drosophila Aurora B), *borr*, and *Incenp* all reduced the percentage of single-signal and paired nuclei at dodeca to 51–66% (*P*<0.05; Figure 3, Table S3). These results complement our identification of *pav* as a pairing promoter in the pilot run, as *pav* encodes a kinesin-like protein required to organize the central mitotic spindle and contractile ring for cytokinesis [64]. Other functionally related pairing promoters include major components of microtubules (αTub84B, βTub56D, βTub85D) and proteins associated with proper mitotic spindle organization (KLP61F, POLO, MARS) (Table S3). Additionally, knockdown of genes associated with chromosome alignment (*chb/mast/orbit* and *cal1*) and cytokinesis (*pbl*, *sti*, *tsr*, *scra*, and *feo*) each increased the number of FISH signals per nucleus as well as the distance between signals (Table S3). FISH targeting euchromatic 16E and 28B revealed a similar reduction in single-signal nuclei from 71 to 31–47% at 16E and from 85 to 35–60% at 28B (*P*<0.05) following *borr*, *pav*, *polo*, *mars*, *chb*, *cal1*, and *sti* RNAi, suggesting these genes are important for pairing at both heterochromatic and euchromatic regions (Figure 3).

Given the well-established role of these genes in mitosis, we sought to determine the state of pairing specifically in interphase as versus that of early mitotic nuclei. FISH targeting dodeca was performed in combination with immunofluorescent labeling of cells with an antibody detecting mitotic phosphorylation of histone H3 on serine 10 (P-H3) to identify and exclude nuclei undergoing mitosis [65]. Our results confirm that pairing was indeed disrupted in interphase (P-H3-minus) nuclei following knockdown of mitotic regulators including *borr*, *ial*, *polo*, *mars*, *chb*, *cal1*, *sti*, as well as for *mcph1*, *dhc64C*, *ida*, and *slmb* (Figure S2). These results suggest that proper spindle assembly, chromosome segregation, and cytokinesis are each independently or collectively important for homologous pairing in interphase nuclei.

### Aneuploidy does not necessarily perturb heterochromatic pairing

Many of the candidate pairing promoters described above are involved in cytokinesis and/or proper chromosome segregation 66,67, which we reasoned might complicate interpretations of their FISH phenotype. For example, we cannot rule out the possibility that increased FISH signals are the result of extra chromosomes caused by aneuploidy. However, as somatic pairing has been shown to accommodate polyploidy in a variety of cell types and tissues [30], [68], extra chromosomal copies may not necessarily be the basis for increased numbers of FISH signals. Indeed, the Kc_167_ cells used in this study are already tetraploid (data not shown and [30]).

**Figure 4:**
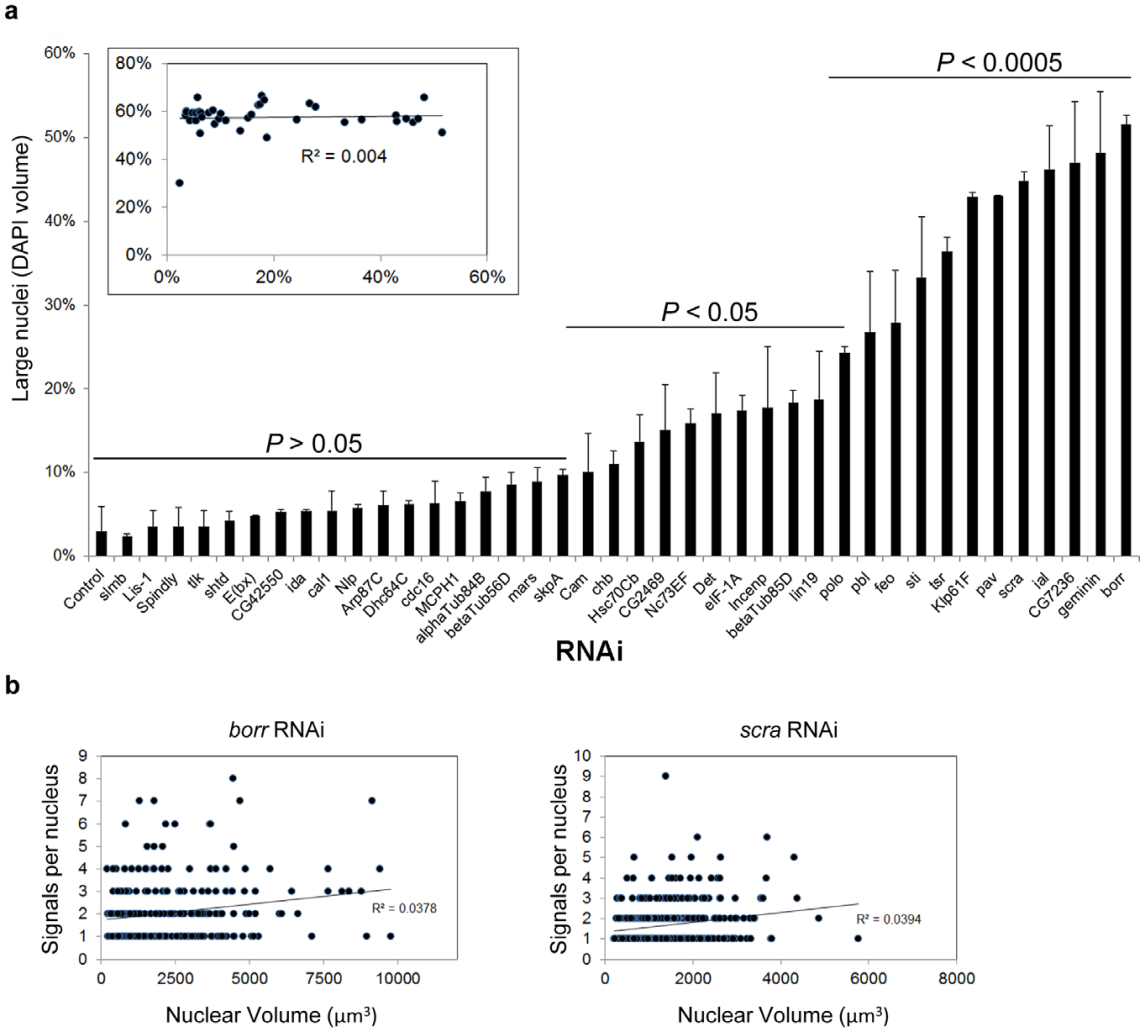
Relationship between nuclear volume and pairing. a, Rank-order plot of the percentage ± SD of large nuclei. A nuclei was considered large if its volume was at or greater than the 95^th^ percentile volume of control cells. X-axis denotes the RNAi target. *P* values were determined by an unpaired *t* test. Inset, the frequency of single-signal nuclei was plotted against the frequency of large nuclei. The coefficient of determination *R^2^* is a measure of how well the data fit a linear regression, with values close to or exactly one representing a perfect fit. As *R^2^* = 0.004, there is no significant correlation between the percentages of paired nuclei and large nuclei. A minimum number of 250 nuclei were scored for each dsRNA. b, The number of FISH signals was plotted against the volume of each nucleus following *borr* and *scra* RNAi. No correlation was found between the degree of unpairing (number of FISH signals) and the size of the nuclei.

To explore the relationship between ploidy and pairing in our system, we recorded changes in nuclear volume, a reasonable proxy for chromosomal content. We found that, after knockdown of *borr*, *CG7236*, *ial*, *scra*, *pav*, and *klp61f*, the volume of >40% of nuclei was at or above the 95^th^ percentile of the control volume of Kc_167_ nuclei (Figure 4), consistent with the frequency of polyploid cells reported in previous studies [66], [67]. Importantly, however, 43% of the candidate pairing promoters did not significantly increase the population of cells with large nuclear volumes upon knockdown (Figure 4, section of graph labeled *P*>0.05), and no correlation was found between the frequency of large nuclei and levels of pairing (*R^2^* = 0.004; Figure 4). Furthermore, despite a >10-fold increase in the frequency of large nuclei following *borr* or *scra* RNAi, no correlation was found between the number of FISH signals and volume for each nucleus (Figure 4). These results argue that RNAi knockdown of candidate pairing promoters has consequences in genome organization during interphase that are independent of ploidy. In line with this observation and discussed further below, our screen failed to recover any member of the cohesin complex, which is required to hold sister chromatids together and maintain proper ploidy [47].

### Cohesin is dispensable for heterochromatic pairing

**Figure 5:**
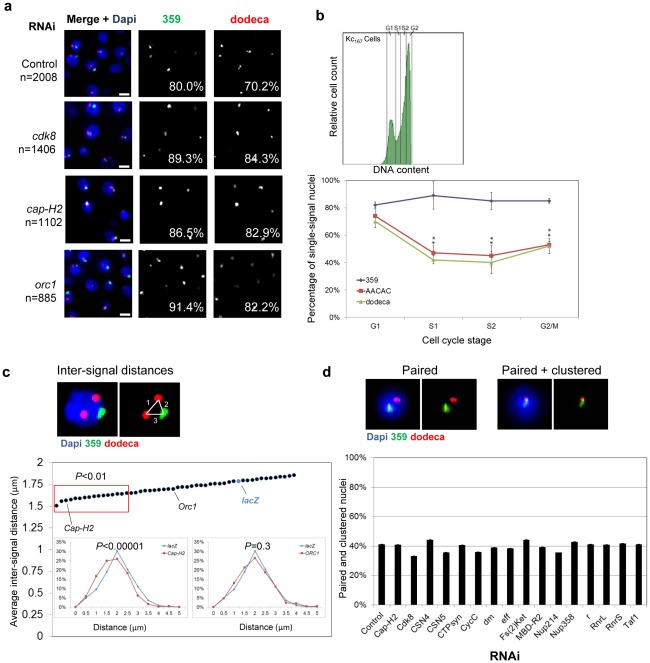
RNAi of candidate anti-pairers enhances heterochromatic pairing frequencies. a, Representative FISH images are shown for RNAi depletion of anti-pairers (*cdk8*, *cap-H2*, and *orc1*), where the number of FISH signals per nucleus is decreased as compared to that of control. Each resulted in a significant increase in the percentage of single-signal nuclei (noted) for both 359 and dodeca (*P*<0.05). n denotes number of nuclei scored. Scale bars equal 5 µm. b, FACS plot (upper) of Kc_167_ cells sorted into G1, early S (S1), late S (S2), and G2/M subpopulations and the percentage of nuclei producing a single FISH signal ± SD when targeting 359, AACAC, and dodeca in each. *P* values were determined by an unpaired *t* test. A minimum number of 100 nuclei were scored for each subpopulation. c, Example of a nucleus in which inter-signal distances were measured. Dot-plot displays the average inter-signal distances per nucleus ± the standard error of the mean (SEM). *Cap-H2*, *ORC1* and *lacZ* RNAi results are noted for reference and red box denotes hits that exhibited a significant shift in the distances per nucleus within the population (*P*<0.01) based on an unpaired *t* test with unequal variance. Insets, relative frequencies of inter-signal distances following *Cap-H2* and *ORC1* RNAi compared to a *lacZ* RNAi control. d, Representative FISH images of a nucleus that produced a single signal for each probe (paired) and a nucleus with partially or fully overlapped 359 and dodeca signals (clustered). No significant difference in clustering levels was observed by this assay following depletion of any anti-pairer as compared to control. Graph displays results for the 16 candidate anti-pairers found to produce a significant reduction in inter-signal distances following RNAi in c (red box). A minimum number of 300 nuclei were scored for each dsRNA.

Considering that separation of sister chromatids should be detectable by FISH and that ∼60% of the Kc_167_ cell population is in G2 (Figure 5), our candidate pairing genes could formally be affecting sister chromatid pairing and, in fact, we had anticipated recovering components of the cohesin complex. Contrary to this expectation, however, no component was identified. RNAi depletion of SMC1, CAP (Drosophila SMC3), and VTD (Drosophila RAD21) resulted in 75±0.2 (*P* = 0.0575), 66±8 (*P* = 0.4359), and 61±9% (*P* = 0.1693) of nuclei with a single dodeca FISH signal; none of these pairing levels differs significantly from the 70±3% observed in controls. As these findings may reflect incomplete knockdown of cohesins, we treated cells with dsRNA for longer time periods (5 and 6 days) and while simultaneously targeting two cohesin subunits. We observed no change in pairing levels in interphase nuclei (data not shown). Furthermore, depletion of Mei-S332/Shugoshin, required to protect pericentromeric cohesion from premature separation [69], also failed to disrupt pairing in our screen (69±0.2%, *P* = 0.6709). We pursued this unexpected finding by assessing knockdown of *rad21* in mitotic nuclei and observed nearly complete chromatid separation in the majority of nuclei (Figure S3), characteristic of sister-chromatid cohesion loss due to efficient knockdown [70]. We therefore propose that sister-chromatid cohesion at pericentromeric heterochromatic regions during interphase can be maintained independent of cohesin or with reduced amounts of cohesin, possibly due to redundancy with pairing interactions between homologs (Joyce, EF, unpublished).

### Genes identified as candidate suppressors of heterochromatic pairing

#### Genes required for the G1-S transition

In addition to candidate pairing promoters, our screen identified 65 candidate anti-pairers, which when depleted decreased the number of FISH signals per nucleus (Table S4). A large fraction (26%) of these proteins promote entry into S-phase or are involved in replication, pointing to the impact of the G1/S transition on pairing (Figure 2). These included the classical positive G1 regulators of the Cdk/E2f pathway, including E2f, CYCLIN E, and DM (Drosophila C-MYC). Depletion of each increased the percentage of single-signal nuclei from 80 to 86–91% at 359 and from 70 to 79–82% at dodeca (*P*<0.05; Table S4). Also identified were the cyclin-dependent kinase CDK8 and corresponding cyclin CYCC, the COP9 signalosome subunits CSN4 and CSN5, and the large and small subunits of ribonucleotide reductase (RNRL, RNRS) that generate nucleotides for replication (Table S4); depletion of each has been shown to inhibit S-phase progression and enrich for a higher G1 population of cells [71]. These results point to the importance of the cell cycle for heterochromatic pairing.

To further investigate the effect of the cell cycle on heterochromatic pairing, we subjected untreated Kc_167_ cells to fluorescence activated cell sorting (FACS) and directly interrogated pairing levels in G1, early S, late S, and G2. In unsorted populations, probes against the 359, AACAC, and dodeca repeats showed pairing frequencies of 80, 42, and 58%, respectively (Figure 1). We found that pairing frequencies at 359 remained unchanged (∼80%) throughout interphase, similar to what has been observed for euchromatic regions [30]. In contrast, we observed higher pairing frequencies at both AACAC (74%) and dodeca (70%) in G1 cells compared to early S (47%, AACAC; 42%, dodeca), late S (45%, AACAC; 40%, dodeca) and G2 cells (53%, AACAC; 52%, dodeca) (Figure 5). A similar pairing dynamic at all three loci was obtained in Drosophila S2R+ cells (Figure S4). These results show that pairing of autosomal heterochromatin is reduced early in replication, similar to that which has been reported to occur *in vivo* [32]. The 359 locus may avoid unpairing during S-phase or, alternatively, unpair temporarily and subsequently pair with faster kinetics than do autosomes, possibly avoiding detection in our analyses of subpopulations. Nevertheless, the reduction in pairing frequencies at the start of S-phase coupled with our identification of G1/S regulators as anti-pairing factors is consistent with this transition representing a critical stage in which pairing interactions are reduced or become more dynamic.

#### Chromosome condensation

We anticipated candidate anti-pairers to include members of the condensin II complex, given its role in antagonizing pairing interactions *in vivo* [31]. Indeed, three subunits of the condensin II complex (Cap-H2, Cap-D3, and SMC2) were identified (Table S4); RNAi depletion of Cap-H2, in particular, increased the percentage of single-signal and paired nuclei from 80 to 87% at 359 and from 70 to 83% at dodeca (*P*<0.05; Figure 5). Importantly, we failed to recover any subunits specific to condensin I, such as *cap-D2*, *cap-G*, or *barr*, the gene encoding Drosophila Cap-H, suggesting the inhibition of heterochromatic pairing is a function of condensin II, not condensin I.

The involvement of condensin II in maintaining the higher-order chromatin state of chromosomes during both mitosis [72], [73] and interphase [74] has suggested a dichotomy between the level of chromatin compaction and the paired state of chromosomes [31]. In line with this model, we also identified HP1a, ORC1, and SLE as anti-pairers (Figure 5; Table S4). HP1a has been shown to concentrate at pericentromeric heterochromatin [75] and is required for the proper compaction of centromeric satellite repeats [76]. Depletion of some ORC subunits also results in condensation defects during mitosis [76], [77]. Likewise, SLE is required for proper compaction of the nucleolus [78]. Therefore, these anti-pairers may include a class of proteins necessary for the intra-molecular compaction of heterochromatic regions.

### Several anti-pairers may also antagonize nonhomologous associations

A reduction in the number of FISH signals per nucleus could represent either a closer alignment of homologs or increased level of centromere/pericentromeric clustering. Such nonhomologous associations could increase the frequency of single FISH signals, yet not necessarily represent homologous pairing. To investigate the extent of heterochromatic clustering in Kc_167_ cells following RNAi of candidate anti-pairers, we measured the inter-signal distances between all signals produced by 359 and dodeca FISH. We reasoned the average distance would serve as a proxy for how coalesced the heterochromatic regions were within each nucleus. In the control, an average inter-signal distance of 1.8 µm per nucleus was observed between all 359 and dodeca signals. When compared to the distribution of distances found in the control population, we found that RNAi of 25% (16/65; see Figure 5d) of the anti-pairers significantly shifted the population towards smaller distances (*P*<0.01, Figure 5), suggesting these genes may have a role in antagonizing nonhomologous heterochromatic associations. Included in these 16 ‘anti-clustering’ candidates are condensin subunit Cap-H2, as well as cell cycle regulators (CDK8, DM, CYCC), proteins important for nucleotide biosynthesis (RNRL, RNRS, CTPsyn, R), and nuclear import (Fs(2)ket, NUP214, and NUP358). The remaining 49 anti-pairers, including HP1a and ORC1, did not significantly change the average inter-signal distances, possibly reflecting the fact that these proteins function specifically in antagonizing homologous interactions. Alternatively, homologous pairing and nonhomologous clustering may be more mechanistically similar than our results indicate; for example, we cannot rule out that pairing and clustering require different levels of activity of the same factors, with pairing being more sensitive to RNAi depletion.

To investigate whether a reduction in distances between nonhomologous sequences following RNAi can cause or contribute to the increased frequency of homologous pairing, we next analyzed the frequency of overlap between the 359 and dodeca FISH signals (Figure 5). We reasoned that if an increased frequency of single FISH signals following RNAi was a direct consequence of heterochromatin clustering, we would also observe an increased frequency in colocalization between nonhomologous sequences (*e.g.* 359 and dodeca). In the control, we found that ∼40% of nuclei that were paired for 359 as well as for dodeca exhibited complete or partial colocalization of the two signals, indicating that the pericentromeric regions of the X and 3^rd^ chromosomes were indeed nonhomologously clustered in a subpopulation of the cells (Figure 5). Surprisingly, RNAi knockdown of each of the 65 candidate anti-pairers including the 16 anti-clustering candidates showed no significant difference in the frequency of colocalization between the two loci. Even of those that exhibited a >10% increased level of homologous pairing, none were found to increase clustering by more than 4% (Figure 5). These results suggest that the increased colocalization of homologous loci following RNAi knockdown of candidate anti-pairers, such as *cap-H2*, cannot be fully explained by heterochromatic clustering. Instead, the decrease in inter-signal distances following RNAi of *cap-H2* and others suggests that these genes may have a role in antagonizing nonhomologous associations in addition to, or perhaps partially contributing to, their role in antagonizing homologous pairing interactions.

### RNAi of a subset of pairing promoters causes Cap-H2–dependent pairing disruption

Considering that pairing levels are sensitive to the level of condensin II activity (this study and [31]), we predicted that some of the pairing promoters might disrupt pairing upon knockdown due to misregulated condensin II. To test this model, cells were depleted for each of the 40 pairing promoters in the presence of dsRNA targeting *cap-H2* and then subjected to FISH targeting dodeca (Table 1, Table S5). These double knockdown experiments, conducted in triplicate in 384-well plates, revealed three subsets of pairing promoters: those completely suppressed, those partially suppressed, and those independent of Cap-H2 co-depletion.

**Table 1:** RNAi of a subset of pairing promoters causes Cap-H2-dependent pairing disruption.

		% of single-signal nuclei^1^		
	dsRNA^2^	blank^3^	+ *cap-H2* 3	*P* value^4^
	*lacZ*	69.1±1.3	82.9±5.2	0.0117
Chromosome structure	*nlp*	65.8±2.2	78.4±1.3	0.001
SCF	*slmb*	29.9±0.3	76.3±0.7	<0.0005
	*lin19*	49.0±7.2	78.7±4.2	0.0035
	*skpA*	56.7±2.5	78.8±2.4	<0.0005
Cytokinesis	*pav*	55.7±4.5	72.1±4.4	0.0211
	*scra*	56.8±2.0	75.2±1.8	<0.0005
	*feo*	61.9±0.0	74.7±0.5	<0.0005
Spindle organization	*polo*	56.3±0.0	74.4±4.5	0.0022
	*βTub85D*	64.7±2.9	74.0±5.1	0.05
	*Arp87C*	59.7±1.7	74.2±6.0	0.02
	*Cam*	58.8±0.5	78.8±2.1	<0.0005
Other	*Nc73EF*	58.6±0.1	79.6±3.4	<0.0005
	*Det*	62.6±1.5	72.6±1.4	0.001

1 Data represent the percentage of single-signal nuclei ± standard deviation for dodeca.

2 N>500 cells for each dsRNA from three trials.

3 No dsRNA (blank) or *cap-H2* dsRNA was added in addition to dsRNA noted in 2^nd^ column.

4 *P* values determined by unpaired *t* test by comparing no dsRNA (blank) to *cap-H2* dsRNA.

#### Condensin-dependent pairing promoters

**Figure 6:**
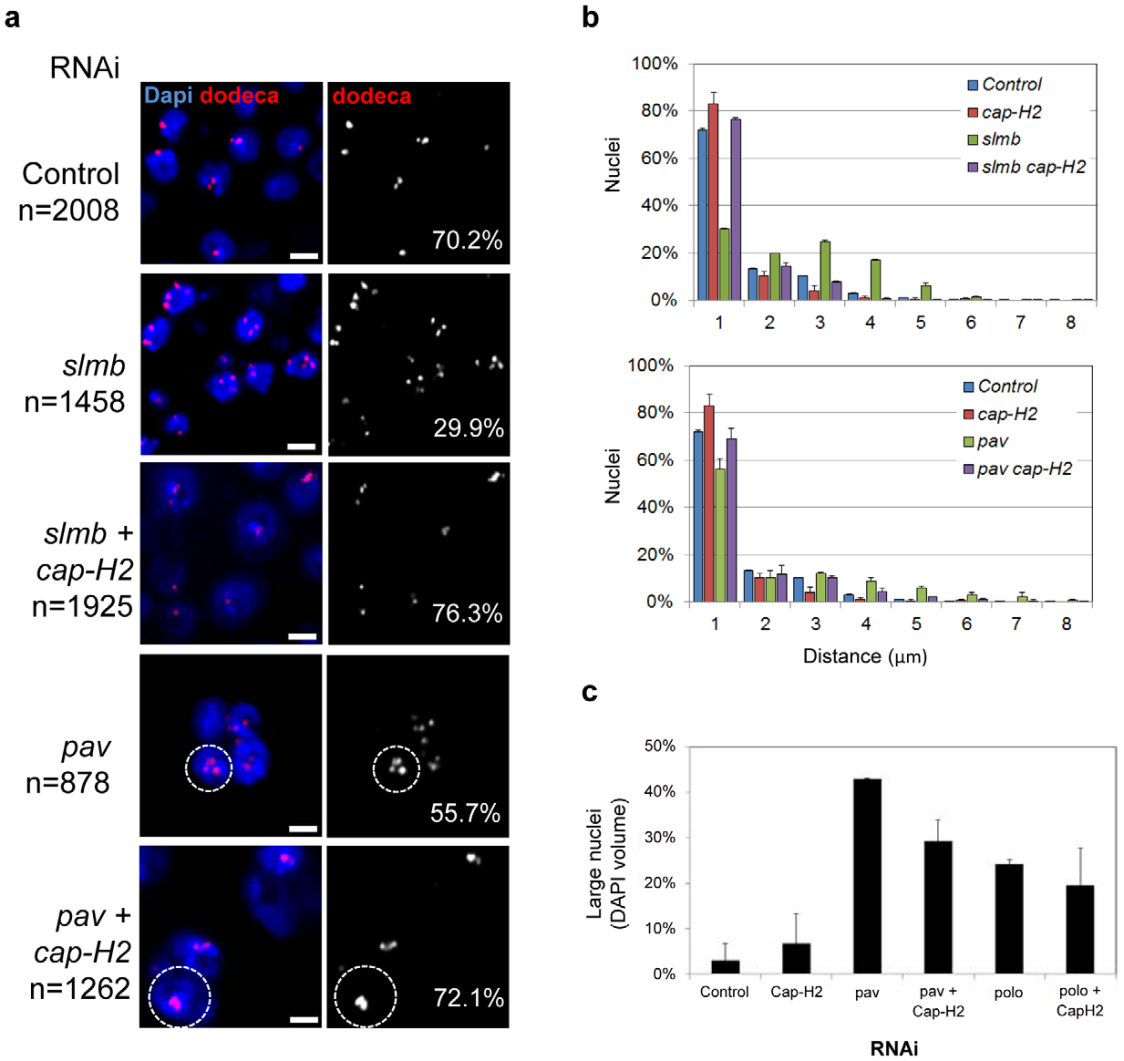
RNAi of a subset of pairing promoters causes Cap-H2–dependent pairing disruption. a, Representative FISH images are shown for RNAi knockdown of candidate pairing promoters *slmb* and *pav*, where the number of single-signal dodeca FISH signals per nucleus (noted) is decreased as compared to *lacZ* RNAi control (*P*<0.05). Co-depletion of Cap-H2 increases the number of single-signal dodeca FISH signals per nucleus (*P*<0.05 compared to *slmb* and *pav* RNAi alone). *pav* RNAi also produces multi-nucleated cells and large nuclei (hashed circles), characteristic of cytokinesis defects that lead to aneuploidy, which are also observed following *pav cap-H2* double RNAi treatment. n denotes number of nuclei scored. Scale bars equal 5 µm. Also see Table 1. b, Relative frequencies of interhomolog distances (unpaired = two signals >1.0 µm apart) based on dodeca FISH ± SD for three tests. Cap-H2 co-depletion also reduces the distances between signals following *slmb* and *pav* RNAi (*P*<0.05), another indication that pairing is restored. c, The percentage of large nuclei ± SD following *pav* and *polo* RNAi in the presence and absence of *cap-H2* RNAi. Although the frequency of large nuclei in *pav cap-H2* is significantly reduced as compared to that of *pav* (*P* = 0.0072), both were significantly increased compared to controls (*P*<0.0001). The frequency of large nuclei in *polo cap-H2* was not significantly different as compared to that of *polo* (*P* = 0.3791). A minimum number of 500 nuclei were scored for each experiment.

Strikingly, we found that 13 (33%) of the pairing hits were completely suppressed by co-depletion of Cap-H2 (Table 1), suggesting that disruption of these candidate pairing promoters perturb pairing in a condensin II-dependent manner. Included in this category was the Drosophila CRP1 encoding gene, *nlp*, previously associated with negatively regulating chromosome condensation [79]; the percentage of nuclei with one dodeca FISH signal was 66% in Nlp-depleted cells but 78% (*P* = 0.001) in *nlp cap-H2* double knockdowns, the latter being similar to levels found following *cap-H2* RNAi alone (*P*>0.2 compared to *cap-H2*; Table 1). Similar suppressions were observed for each component of the SCF complex; knockdown of *slmb*, *lin19*, and *skpA* reduced the frequency of single-signal nuclei to 30, 49, and 56%, respectively, whereas co-depletion of Cap-H2 increased those levels to 76, 79, and 79%, respectively (*P*<0.005; Figure 6, Table 1). Additionally, we found that the reduced nuclear volumes present in SLMB-depleted cells (587±256 µm^3^) were suppressed by Cap-H2 co-depletion (1,010±541 µm^3^, *P*<0.0001). Thus, these results reveal a novel genetic interaction between the SCF ubiquitin-ligase and condensin II complexes that is important for nuclear organization.

Additional dsRNAs whose FISH phenotypes were dependent on Cap-H2 included those targeting genes known to be involved in cytokinesis (*pav*, *scra*, and *feo*) and mitotic spindle organization (*polo*, β*Tub85D*, *Arp87C*, and *Cam*) (Figure 6, Table 1). Importantly, the high levels of large nuclei and multi-nucleated cells observed following *pav* and *polo* RNAi was not completely suppressed in *cap-H2* double knockdown experiments (Figure 6). Our examination of pairing within multi-nucleate cells further revealed that individual nuclei often produced a single large FISH signal, indicating close alignment of homologs despite the increased chromosomal content (Figure 6). This separation of pairing and large nuclei phenotypes confirms our observation that pairing can accommodate larger nuclear volumes and extra chromosomal copies.

#### Condensin-independent pairing promoters

The remainder of the pairing hits were either partially (12, 30%) or completely independent (15, 37%) of Cap-H2 co-depletion (Table S5), perhaps revealing a second, condensin-independent pathway important for pairing. Those that were partially suppressed typically restored pairing to control levels, which are, however, significantly reduced as compared to that produced by *cap-H2* RNAi. Proteins corresponding to these hits are involved in chromosome organization or alignment (*e(bx)* and *tlk*) and spindle organization (*αTub84B* and *mars*) and include members of the APC (*shtd* and *cdc16*). We cannot rule out the possibility that some wells experienced inefficient Cap-H2 depletion, although we consider this to be unlikely given the low variability between three replicate tests (Table S5).

Those dsRNAs whose effects were unchanged in the presence of *cap-H2* RNAi targeted genes that encode components of the CPC (*ial* and *Incenp*) and genes associated with MTOC function (*mcph1* and *dhc64C*). dsRNAs targeting genes necessary for cytokinesis (*pbl*, *sti*, and *tsr*) and chromosome alignment (*klp61f* and *cal1*) were also all found to elicit their pairing effects independent of Cap-H2. Thus, these data argue that pairing can be influenced by a complex network of genes, including those that function through condensin II and those that do not.

## Discussion

**Figure 7:**
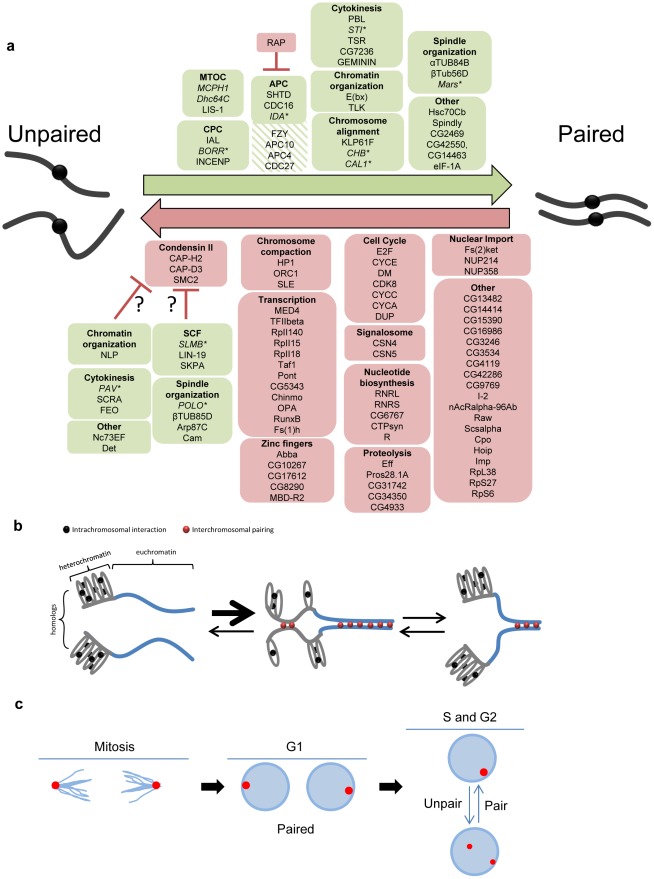
Pairing models involving both candidate pairing promoting and anti-pairing factors. a, Summary of candidate pairing factors identified in the screen. Green boxes denote candidate pairing promoters (hatched green were those identified only in the primary screen) and red boxes denote candidate anti-pairers. A representative sampling of pairing promoters were tested (italicized) and found (asterisk) to be important for euchromatic pairing. Proteins are grouped based on either a known function or localization pattern. Candidate pairing promoters found to elicit RNAi phenotypes dependent on Cap-H2 are presented as potential condensin II regulators (question marks). Note one dsRNA targets both CG42550 and CG14463 (separated by comma). b, Model for how compaction and intrachromosomal interactions compete with homolog pairing. Although all chromosomal regions may transiently unpair prior to or during S-phase, homolog pairing (red circles) of heterochromatic centromeric regions (grey lines) may be in competition with intrachromosomal interactions (black circles), causing pairing to occur less often, more slowly, or with less stability than homolog pairing of less compacted euchromatic regions (blue lines), where the paucity of repeated sequences reduces the likelihood of intrachromosomal interactions. This figure is not meant to imply a causal or dependent relationship between heterochromatic and euchromatic pairing, although such a relationship may exist. c, Model for pairing through the cell cycle. Proper spindle formation and chromosome segregation during anaphase/telophase of mitosis may bundle centromeric heterochromatic regions to spindle poles and directly facilitate or accelerate homolog recognition. Such interactions would then be maintained through G1. During S-phase, however, the pairing of regions is perhaps more dynamic, becoming antagonized and then re-paired subsequently. In this case, not all pairing interactions would be reestablished until the following mitosis. Euchromatic pairing is not depicted.

In this report, we introduce a high-throughput FISH technology that enabled a genome-wide RNAi screen for factors involved in somatic homolog pairing. We identified both candidate pairing promoting genes as well as candidate anti-pairing genes, supporting the idea that homologous pairing is mediated by a balance of factors with opposing functions (Figure 7). As discussed below, these results also led to insights into the relationships between somatic pairing and the cell cycle and condensed state of chromosomes.

### Somatic pairing may involve multiple mechanisms

Our data are consistent with different regions of the Drosophila genome exhibiting different levels and stabilities of pairing [30], [33], [43], [44], suggesting multiple independent mechanisms and/or different regional sensitivities may contribute to the overall somatic pairing of homologous chromosomes. For example, heterochromatin and euchromatin display different pairing frequencies, have different cell cycle dynamics and, as revealed by studies of Top2, MCPH1, and Dhc64C, might have different genetic requirements (this study and [30], [32], [44]). In fact, the existence of independent pairing mechanisms might explain why we were unable to completely abolish pairing in our screen; ∼30% of nuclei remained paired following dsRNA treatment targeting of our strongest pairing promoter, *slmb*, although, of course, this result could also be a consequence of incomplete RNAi. However, it is important to note that of the 11 representative heterochromatic pairing promoting genes we tested, 9 are important for homologous pairing of euchromatic regions as well. Therefore, the pairing of heterochromatic and euchromatic regions may be established by the same mechanism(s) and maintained independently, vice versa, or, perhaps, achieved through overlapping forces, with the potential of each contributing *in cis* to the proximity of the other.

Our work further indicates that even amongst heterochromatic regions, different loci may exhibit different stabilities of pairing. For example, pairing frequencies at the 359-bp repeat locus on the X-chromosome are higher compared to that of the autosomal heterochromatic repeated sequences AACAC and dodeca (∼80% compared to ∼50%), suggesting that pairing of 359 may be more difficult to disrupt. Indeed, only 16% of the pairing hits significantly affected pairing at this locus, and 359 was the only heterochromatic region tested that did not reveal a drop in pairing levels during S-phase. We reason the more robust pairing of 359 could be due to structural features, such as the large nature of the repeated region, estimated to be ∼11 Mb in length, and/or the acrocentric nature of the X-chromosome. Alternatively, the proximity of the X-linked rDNA gene cluster to 359 may contribute to pairing via independent forces, as the rDNA locus is associated with pairing of the X and Y chromosomes during male meiosis [80]–[82].

### A mechanism for preventing pairing?

Our most striking result was the abundance of candidate anti-pairers which, similar to Cap-H2 [31], increased the frequency of single-signal nuclei when knocked down (Figure 7). If exclusively or especially effective at heterochromatic regions, anti-pairing could explain why pairing frequencies are reduced at heterochromatic regions compared to that of euchromatic [30]. Thus, the temporal and spatial regulation of anti-pairing proteins could be an important aspect of pairing-dependent processes.

The advantages of preventing heterochromatic pairing might include safeguarding the genome against aberrant repair or mitotic recombination. Specifically, the unpairing of similar heterochromatic sequences between homologous and heterologous chromosomes would preclude either from being used as templates for repair and therefore prevent loss-of-heterozygosity or chromosome rearrangements, respectively. The anti-pairing activity of HP1a is particularly interesting in this light, as HP1a has been proposed to contribute to the prevention of aberrant repair between nonhomologous chromosomes in heterochromatin by relocalizing broken sites outside the heterochromatic domain [83]. Our observations suggest that HP1a may facilitate DSB relocalization by unpairing homologs, which could potentially increase chromosome mobility. Homolog separation would further ensure accurate repair by favoring use of the sister chromatid as the repair template. Anti-pairing could become especially important during replication, as spontaneous damage can result from the passage of replication forks through highly repetitive DNA [84]. Thus, this viewpoint is consistent with the reduced heterochromatic pairing we observed during and immediately after S-phase. Indeed, a large quantity of anti-pairers included cell cycle regulators required for entry into S-phase (*e.g. e2f* and *cdk8*), suggesting that homolog unpairing is functionally coupled to the progression of the cell cycle.

Anti-pairing may also be a potent means to globally, locally, and/or transiently control gene expression through the cell cycle. For example, it may be used to inhibit cross-communication between alleles that are physically paired [30], [31] and may explain why, despite intimate pairing in Drosophila, reports of transvection are relatively rare throughout the genome. It could even constitute a conserved form of gene control, a concept in line with studies of human renal oncocytomas, where the paired state of the q arm of Chromosome 19 is correlated with misexpression [85].

Finally, our screen suggests that the mechanism of anti-pairing involves the maintenance of higher order chromosome structure, as it recovered several components of the condensin II complex and ORC1, in addition to HP1a, as anti-pairers. The connection between anti-pairing and condensin II is particularly intriguing, given the role of the latter in chromosome compaction. How might anti-pairing and compaction be mechanistically linked? One possibility is that the forces of compaction drive the formation of unpaired loops [31]. Alternatively, anti-pairing may drive compaction, where the formation of intermittent unpaired loops pull flanking chromosomal regions closer together.

Interestingly, the human ORC complex has been implicated in HP1a recruitment to heterochromatin [76], and both the ORC complex and HP1a are required for proper compaction of centric heterochromatic satellite repeats [86]–[89]. These observations are line with compaction being a mechanism by which heterochromatic pairing is inhibited, which could be facilitated by the capacity of arrays of repeated sequences to fold back on themselves and interact intrachromosomally as versus between homologs [30] (Figure 7). The need to preclude heterochromatic pairing, and hence aberrant repair or recombination, may even provide an explanation for why heterochromatin remains compacted through interphase. Intriguingly, ORC1 depletion leads to centromeric clustering in human cells [76], possibly reflecting a conserved role in antagonizing interchromosomal interactions.

### A subset of pairing hits are dependent on Cap-H2 activity

Given the role of condensin II in anti-pairing, it is perhaps not surprising that many of the candidate pairing promoters we identified had RNAi phenotypes that were dependent on Cap-H2 activity. This ‘condensin-dependent’ subset of pairing promoters may facilitate pairing indirectly through controlling condensin II activity (Figure 7). For example, of those completely suppressed by reduced Cap-H2 activity were three members of the SCF complex, *slmb*, *lin19*, and *skpA*. Considering that SCF functions as an E3 ubiquitin-ligase [58], [59], it may support pairing by controlling the level of condensin II during interphase and targeting this complex for degradation. Consistent with this model, *slmb* RNAi also resulted in smaller nuclear volumes, a phenotype dependent on Cap-H2 and characteristic of hypercondensation. Alternatively, the role of SCF in pairing may be linked to its role in cell cycle regulation, with knockdown causing an enrichment of stages where pairing is normally reduced.

Additional dsRNAs whose FISH phenotypes were dependent on Cap-H2 included those targeting genes known to be involved in mitotic spindle organization or cytokinesis, such as *polo* and *pav*. Interestingly, while Cap-H2 co-depletion suppressed the pairing phenotype following *polo* and *pav* knockdown, only a mild reduction in the frequency of large nuclei was observed. Thus, the increased nuclear volumes and multi-nucleated cells (both characteristic of cytokinesis defects) in the double knockdowns confirms that pairing can accommodate larger nuclear volumes and, likely, extra chromosomal copies. Although the relationship between *cap-H2* and these pairing promoters remains to be elucidated, our findings argue that these factors may lead to a disruption in pairing by modulating condensin II activity and/or inhibiting the activity of proteins necessary for decondensation at the end of mitosis.

We note an alternative model in which the consequences of depleting pairing promoters can be countered by the loss of condensin II. For example, the role of condensin II in resolving DNA catenations suggests that pairing may involve DNA catentation and, if so, *cap-H2* RNAi may suppress unpairing simply by precluding paired homologs from decatenating. Considering that mitotic spindle forces are required for the resolution of DNA catenations [90], this interpretation suggests that co-oriented and catenated homologs attached to the same spindle pole might remain catenated and therefore paired into the next cell cycle by escaping antagonizing spindle forces. In light of this, those gene knockdowns that disrupt spindle stability (*e.g*. *pav* RNAi) could create new antagonizing forces against homologous chromosomes and thus aberrantly remove any residual catenations or pairing interactions. In the absence of condensin II, however, the accumulation of DNA catenations between homologous chromosomes may enhance pairing and prevent homolog separation.

### Potential models for somatic pairing

Of the candidate pairing promoters, 27 had RNAi phenotypes that were not dependent or only partially dependent on Cap-H2 activity (Figure 7, Table S5). This ‘condensin-independent’ class includes members of the APC, components of the CPC, and proteins involved in spindle organization, chromosome alignment, and cytokinesis. Perhaps the most surprising feature of these results is the level of conservation among these genes; 25 out of 27 of these candidate pairing promoters have putative human orthologs (Table S5), possibly suggesting that eukaryotes have generally retained the mechanism and therefore ability to pair homologs.

Our identification of mitotic regulators is consistent with a critical step of pairing occurring during mitosis, with anaphase and/or telophase being of particular import. Although pairing may be disrupted at the onset of anaphase [43], [44], the drawing of centromeric regions to spindle poles during late anaphase/telophase could directly facilitate or accelerate the homolog interactions by bundling heterochromatic regions into a relatively small volume (Figure 7). This chromosomal arrangement, in which centromeres point toward poles with telomeres dragging behind may resemble a Rabl configuration, which has been proposed to promote homolog pairing by reducing the nuclear space in which chromosomes search for their homologs [44], [91], [92]. This idea is supported by our identification of pairing promoters essential for focusing microtubules to spindle poles during anaphase (*dhc64C*, *lis-1*, and *mcph1*; [93], [94]) and many genes that encode proteins necessary for spindle assembly, chromosome alignment, and/or the metaphase-anaphase transition; disruption of each would impair the proper bundling of heterochromatic regions. With the exception of *dhc64C* and *mcph1*, these genes were also found to be important for euchromatic pairing, suggesting the pairing of chromosome arms could be facilitated and/or stabilized by a similar mitosis-driven mechanism, possibly extending, although not necessarily linearly, from both pericentromeric and interstitial heterochromatin. A complete understanding of pairing, however, will require a screen for pairing factors wherein the FISH assay targets euchromatic loci directly to determine whether there are pairing factors that are essential only at euchromatin.

An additional, yet not mutually exclusive, model proposes that at least some pairing promoters function directly in the maintenance of homolog pairing during interphase. For example, kinesin Klp61F, Dynein motor protein Dhc64C, and microtubule binding protein CHB also localize to cytoplasmic interphase microtubule arrays [93], [95], [96]. Intriguingly, cytoplasmic microtubule-based movement (involving Dynein) has a wide-spread role in ensuring proper and timely homolog pairing during meiosis, presumably by inhibiting incorrect nonhomologous associations, which as we discussed above, may be in competition with homologous pairing [97].

Lastly, as we identified CPC components INCENP and IAL (Drosophila Aurora B) as condensin-independent pairing promoters, an intriguing parallel to our work may be the discovery of DNA tethers, coated with INCENP and Aurora B, that connect and mediate the accurate segregation of chromosome fragments at mitosis [98] as well as achiasmate homologous chromosomes in Drosophila female meiosis [99]. These DNA linkages may be a general feature of Drosophila chromosomes and share genetic properties with somatic pairing mechanisms. Therefore, additional hits identified in this screen may also prove to be important for the pairing and accurate segregation of homologous chromosomes during meiosis.

In sum, this study indicates that somatic homolog pairing may be mediated by antagonistic mechanisms, possibly involving >100 genes, many of which are highly conserved throughout higher eukaryotes. Excitingly, a number of these genes have also been identified as pairing factors through a whole-genome screen examining localization of the MSL dosage compensation machinery in Drosophila (J. Bateman and E. Larschan, personal communication). Our work also brings support to long-standing observations correlating stages of the cell cycle to differing degrees of homolog pairing [43]–[46] and further provides a genetic framework suggesting that progression through the cell cycle can facilitate, adjust, and thus control pairing-dependent processes. Indeed, cell cycle progression, per se, may constitute a potent means by which cells control gene regulatory mechanisms that rely on interchromosomal interactions, with prolonged duration or arrest in G1, S, G2, or mitosis enhancing or suppressing such interactions [30]. Homolog pairing may even, in turn, exert some control over cell cycle progression. A fuller picture of pairing, however, awaits a parallel screen for factors involved in pairing at euchromatic loci in Drosophila as well as studies of pairing, in general, in other organisms. Finally, this report describes a technology for high-throughput FISH, which can be widely applied to the analysis of chromosome positioning and nuclear organization.

## Materials and Methods

### Cell culture

Kc_167_ cells [100], [101] obtained from the Drosophila Genome Resource Center were grown at 25°C following standard protocols. Cultures were grown in sterile filtered Schneider's medium (GIBCO) supplemented with heat-inactivated fetal bovine serum (FBS, to a final concentration of 10% v/v) and penicillin–streptomycin (50 units/mL penicillin, 50 µg/mL streptomycin; GIBCO). To ensure that experiments were done with log-phase cells, active cultures were split at a 1∶5 ratio, cultured for 3–4 days, and then passaged at 2–4×10^6^ cells/mL prior to the analyses.

### Generation of FISH probes

Oligo probes for the 359, AACAC, and dodeca heterochromatic repeats [54], [102] were synthesized with a 5′ Cy5, Tye3, and FAM488 fluorescent dye, respectively, by Integrated DNA Technologies (IDT). Probe sequences were designed to be relatively small in length (15 to 35 bases) for efficient nuclear integration and have melting temperatures >70°C to withstand stringent wash conditions. Probe specificity was tested by hybridization to metaphase spreads (data not shown). Those sequences found to produce the most robust signal at the lowest concentration with highest level of specificity were selected for future analyses. The sequences are as follows: Cy5-359: Cy5-GGGATCGTTAGCACTGGTAATTAGCTGC, Ty3-AACAC: Tye3-AACACAACACAACACAACACAACACAACACAACAC, and FAM488-dodeca: FAM488-ACGGGACCAGTACGG. Oligo probes were resuspended in 1×TE at 100 µM concentration and stored at −20°.

DNA probes to 16E and 28B were synthesized according to standard protocols. Bacterial artificial chromosome BACR17D02 RP98-17D2 (AC012163; AE003507) corresponding to 16E1–16E2 (abbreviated as 16E) and P1 plasmids (Berkeley Drosophila Genome Project) containing cloned Drosophila genomic DNA corresponding to chromosomal regions 28B1–28B2 (abbreviated as 28B; DS01529; [44]) were synthesized and labeled by nick translation/direct labeling (Vysis) following the manufacturer's protocol.

### RNAi

Synthesis of dsRNA and application of RNAi to cells was carried out according to published protocols [101]. Control cells were treated with a blank of deionized water or, when noted, dsRNA targeting *lacZ*. Cells were fixed 4–5 days after treatment.

### Standard FISH protocol

Our standard FISH protocol on slides was adapted from previously published protocols [30], [102], [103] and involved the following steps: Cells from log-phase cultures were adhered to lysine-treated glass slides for 1 hr. Slides were then gently washed with PBS (pH 7.2), fixed for 5 minutes with 4% formaldehyde in PBS at room temperature (RT), washed in 2×SSCT (0.3 m NaCl, 0.03 m sodium citrate, 0.1% Tween-20) for 5 minutes at RT, and washed in 2×SSCT/50% formamide for 5 minutes. Pre-denaturation steps were carried out as follows: 2×SSCT/50% formamide at 92° for 3 minutes and then 60° for 20 minutes. DNA probe in hybridization buffer (20% dextran sulfate/2×SSCT/50% formamide) was then added to the slides, covered with a coverslip, and denatured on a heat block in a water bath set to 92° for exactly 3 minutes, after which slides were transferred to a humidified chamber and incubated overnight at RT. Coverslips were then removed while the slides were being washed (2×SSCT at 60° for 10 minutes). For FISH with euchromatic probes (either P1 or BAC generated), an additional wash at RT in 0.2×SSC was conducted for 10 minutes. A final RT wash in 2×SSCT was then done for 5 minutes, after which Slowfade with DAPI (Invitrogen) was added. Coverslips were applied and sealed to the slides with nail polish. Images were collected using an Olympus IX81 fluorescence microscope with a 60×, N.A. 1.35 lens. Nuclei were imaged by collecting optical sections through the entire nucleus. The data are shown as maximum projections; however, the analysis of the images was performed by examining one section at a time.

### 384-well FISH protocol

A 384-well plate containing dsRNA was centrifuged at 1200 RPM for 2 minutes. Log-phase Kc_167_ cells (grown for 3 days) were centrifuged (1200 RPM for 5 minutes), counted, and diluted in FBS-free media to 1×10^6^ cells/mL. Sterile, autoclaved Wellmate tubing was purged with sterile PBS in a tissue culture hood. After the Wellmate was primed with the diluted cells, 10 µL were added to each well. The plate was then spun (1200 RPM, 2 minutes) and incubated in a 25°C incubator for 30 minutes. With freshly primed sterile tubing, regular FBS-containing Schneider's media was added to each well (30 µL/well) and the plate was then spun (1200 RPM, 2 minutes) before being transferred to a humidifying chamber in a 25°C cell culture incubator. Four (primary screen) or five (validation screen) days after dsRNA treatment, the plates were removed from the incubator. The cell media was aspirated and, with a primed Wellmate, wells were quickly washed with PBS (60 µL/well), which was then immediately aspirated. Plates were incubated with 4% Formaldehyde (30 µL/well) for 5 minutes, aspirated, and quickly rinsed (30 µL PBS/well), then washed with 2×SSCT (80 µL/well) for 5 minutes. Then, plates were washed with 50% formamide/2×SSCT (80 µL/well) for 5 minutes. The plates were double-sealed with adhesive aluminum seals, pre-denatured by being floated in a 91°C waterbath for 3 minutes, 60°C waterbath for 20 minutes, and allowed to cool to room temperature.

Probes were prepared in 10 mL of Hybridization Buffer (20% dextran sulfate/2× SSCT/50% formamide) per plate [the 100 µM stock solution of oligo probes (see above) was diluted 1∶10,000 and 1∶5,000 for FAM488-dodeca and Cy5-359, respectively]. The plate was aspirated, after which probe mix was added to each well (20 µL/well). The plates were again double-sealed with aluminum adhesive seals, centrifuged (1200 RPM for 2 minutes), and denatured in a 91°C waterbath for 20 minutes. Hybridization was conducted for 30 minutes at 45°C by floating the plate in prewarmed 45°C wash buffer (50% formamide in 2×SSCT) in a Tupperware within an incubator. The plate was washed by being submerged in 45°C wash buffer while having its seal removed, allowing buffer to wash immediately into the wells. The plates, still submerged in 45°C wash buffer, were then placed on a slow moving shaker. Buffer was vigorously “flicked” out of the wells after 5 minutes and again after 20 minutes, being quickly resubmerged after each. The plate was aspirated and washed with room temperature 50% formamide/2×SSCT (80 µL/well) for 5 minutes. Hoechst was diluted 1∶1,500 in 2×SSCT and added to each well (30 µL), after which the plate was incubated for 5 minutes. The plate was then washed twice for 10 minutes each with 2×SSCT (60 µL/well). The plate was then sealed with a clear adhesive seal and centrifuged (1200 RPM for 2 minutes). To ensure optimal imaging, all plates were prepped and imaged in the same day. For automated microscopy, the cells were imaged with an Evotec Opera Confocal Screening Microscope (Perkin-Elmer) with a 63× water immersion lens. 10 images per well were acquired, each of which were autofocused prior to taking a single optical section through the nuclei. Note that the Opera system had limits as to how many images could be taken per well. Considering the short depth of nuclei within adhered Drosophila cells and the brightness of our FISH signals, we chose to take 10 autofocused images per well without Z-slices to maximize the number of cells being assayed.

### Automated data analysis

All the images acquired in this screen were analyzed automatically using a customized algorithm developed in MATLAB. The analysis was carried out in three main steps: nuclear segmentation, FISH foci identification, and cell classification/scoring. In the nuclear segmentation step, the DAPI image was first smoothed and background corrected. The corrected image was then segmented by applying a threshold determined by Ridler-Calvard method [104]. To correct for under-segmentation caused by cell clustering, large clusters of nuclei were first identified, and then processed through a shape-based watershed to divide individual nuclei apart. In the second step, each nucleus was analyzed individually to identify foci in both red (dodeca, pseudocolored) and green (359, pseudocolored) channels. To achieve this, an image patch was cropped out of smoothed green/red image based on nuclear mask from the first step. For each nucleus, the median value and standard deviation of intensity were determined in the nuclear region for both red and green channels. A threshold, which is two standard deviations higher than the median value, was then applied to the red/green image crop to pick up all the bright spots in the nuclear region in both channels. The bright spots were then filtered based on size criteria to prevent false detection caused by background noise. Following foci identification, we classified/scored cells using three different approaches. In approach #1, we sorted all cells into six different groups based on the number of foci they contained in each channel, namely 0, 1, 2, 3, 4, ≥5 foci. In approach # 2, we measured the pairwise distances between foci of the same color to further analyze pairing as well as for both colors to investigate clustering. In the third approach, we identified cells with colocalized foci of different colors (one red and one green) by checking whether there were any overlapping pixels between the foci detected from two different channels within any given nucleus. Finally, we tried to identify those dsRNAs from our hit list that caused an abnormal level of polyploid cells due to failed cell division. For this purpose, we filtered all the cells based on their size using a cut-off value determined from the largest 95^th^ percentile cell sizes of control wells (data not shown).

### Criteria for primary and validation screen cut-offs

In the primary genome-wide screen, a dsRNA was considered a ‘pairing promoting’ hit if, in both replicate plates, the dsRNA either decreased the percentage of nuclei with a single FISH signal to a *z*-score of ≤−2.0 or increased the percentage of nuclei containing two, three, or four foci to a *z*-score of ≥2.0. 374 dsRNAs were considered hits using these criteria. Importantly, using these cut-offs, greater than 90% of positive control wells seeded with dsRNA targeting *pav* or *cap-H2* resulted in the expected increase or decrease in FISH signals per nucleus. In the validation screen, however, we sought to identify the strongest hits and, therefore, only listed dsRNAs that significantly (*P*≤0.05) decreased the percentage of nuclei with a single FISH signal as compared to control cells. This created a much more stringent cut-off and reduced the number of gene hits to 40.

For dsRNAs that produced an ‘anti-pairing’ phenotype in the primary screen, each replicate plate produced an increase in the percentage of nuclei with a single FISH signal to a *z*-score of ≥2.0. Similarly, the criterion used for hits in the validation screen was those dsRNAs that significantly (*P*≤0.05) increased the percentage of nuclei with a single FISH signal as compared to control cells.

### Immunofluorescence

Primary antibody against phosphohistone H3 (P-H3; rabbit used at 1∶100; Epitomics) was used for immunofluorescence in a PBS buffer following FISH reactions. A Cy3-conjugated anti-rabbit secondary antibody (Jackson ImmunoResearch Laboratories) was used at 1∶165 according to the manufacturer's instruction.

### Metaphase spreads

Kc_167_ cells were grown in six-well plates, pipetted onto slides, exposed to hypotonic solution (1% Sodium Citrate) for 30–45 minutes and then fixed in 3 Methanol: 1 Acetic acid. Cells were then dried for a few minutes and DNA was stained with DAPI.

### Cell sorting

Fixed cells were RNAsed (1 mg/mL), incubated with a 2 µM solution of Propidium Iodide (PI), and sorted on a Becton Dickinson FACSAria. The cells were then adhered to lysine-coated slides for 2–3 hours, and then subjected to FISH. We confirmed that cells had been successfully sorted into G1, early S, late S, and G2 subpopulations by assessing nuclear DNA content as determined by DAPI staining (data not shown).

## Supporting Information

Figure S1 Correlation between 359 and dodeca pairing following RNAi knockdown of pairing promoters. The results from RNAi of all 40 pairing promoters are plotted. X-axis is the percentage of single-signal nuclei at 359. The Y-axis is the percentage of single-signal nuclei at dodeca. The coefficient of determination *R^2^* = 0.3586 represents significant fit of the data to a linear regression, suggesting that pairing levels between the two chromosomal regions are correlative. A minimum number of 250 nuclei were scored for each dsRNA.
(TIF)

Figure S2 Pairing promoters are important for pairing in interphase nuclei. a, Representative image showing both interphase (PH-3-minus) and mitotic (PH-3-positive) nuclei with dodeca FISH. b, Following depletion of 11 representative pairing promoters, the percentage of single-signal nuclei was significantly decreased compared to control (*P*<0.05). Error bars denote SD. A minimum number of 100 nuclei were scored for each dsRNA.
(TIF)

Figure S3 RAD21 depletion leads to premature sister chromatid separation during mitosis. Chromosomes from control metaphase cell with paired sister chromatids and from a RAD21 RNAi metaphase cell clearly showing separated sister chromatids.
(TIF)

Figure S4 Heterochromatic pairing through the cell cycle in S2R+ cells. FACS plot of S2R+ cells with four gates for G1, early S (S1), late S (S2), and G2 phases of the cell cycle. The frequency ± SD of paired nuclei when targeting 359, AACAC, and dodeca in the G1, S1, S2, and G2 subpopulations. Asterisks denote a significant reduction in paired nuclei at each locus compared to that of G1 cells (*P*<0.05). A minimum number of 100 nuclei were scored for each subpopulation.
(TIF)

Table S1 Comparison of pairing levels under different control conditions.
(XLSX)

Table S2 Pairing candidates isolated in primary screen. dsRNAs listed are those that produced a significant *z-*score in the primary screen. Plate and well location are noted along with dsRNA amplicon and target gene (if one is annotated). %1 359 and %1 dod denote the percentage of nuclei with a single 359 or dodeca focus, respectively. Corresponding *z-*scores are noted.
(XLSX)

Table S3 Validated candidate pairing promoters. dsRNA amplicon, target gene, and well location are noted along with total cell count (Cell_cnt), the percentage of large nuclei (Large_nuclei), and the percentage of nuclei with one, two, three, four, and five or more FISH foci for 359 and dodeca. The percentage of nuclei with a single focus for both 359 and dodeca (1R1G_nuclei) as well as the subset of these that exhibit colocalization (1R1G_touch_nuclei) are also noted. Standard deviations (stdev) from three replicate tests are presented to the right of each parameter.
(XLSX)

Table S4 Validated candidate anti-pairers. dsRNA amplicon, target gene, and well location are noted along with total cell count (Cell_cnt), the percentage of large nuclei (Large_nuclei), and the percentage of nuclei with one, two, three, four, and five or more FISH foci for 359 and dodeca. The percentage of nuclei with a single focus for both 359 and dodeca (1R1G_nuclei) as well as the subset of these that exhibit colocalization (1R1G_touch_nuclei) are also noted. Standard deviations (stdev) from three replicate tests are presented to the right of each parameter.
(XLSX)

Table S5 Cap-H2–independent pairing promoters. Data are presented for the pairing promoters whose RNAi phenotypes were either partially or completely independent of Cap-H2 co-depletion. dsRNA target genes and thier putative human orthologs are noted as well as the percentage of nuclei with a single dodeca FISH signal in the presence and absence of Cap-H2 RNAi.
(XLSX)
