# Supplementary figures and images for: Identification of Genes That Promote or Antagonize Somatic Homolog Pairing Using a High-Throughput FISH–Based Screen
Source: PLoS Genet. 2012 May 10;8(5):e1002667. doi: 10.1371/journal.pgen.1002667 (PMC3349724; doi:10.1371/journal.pgen.1002667)

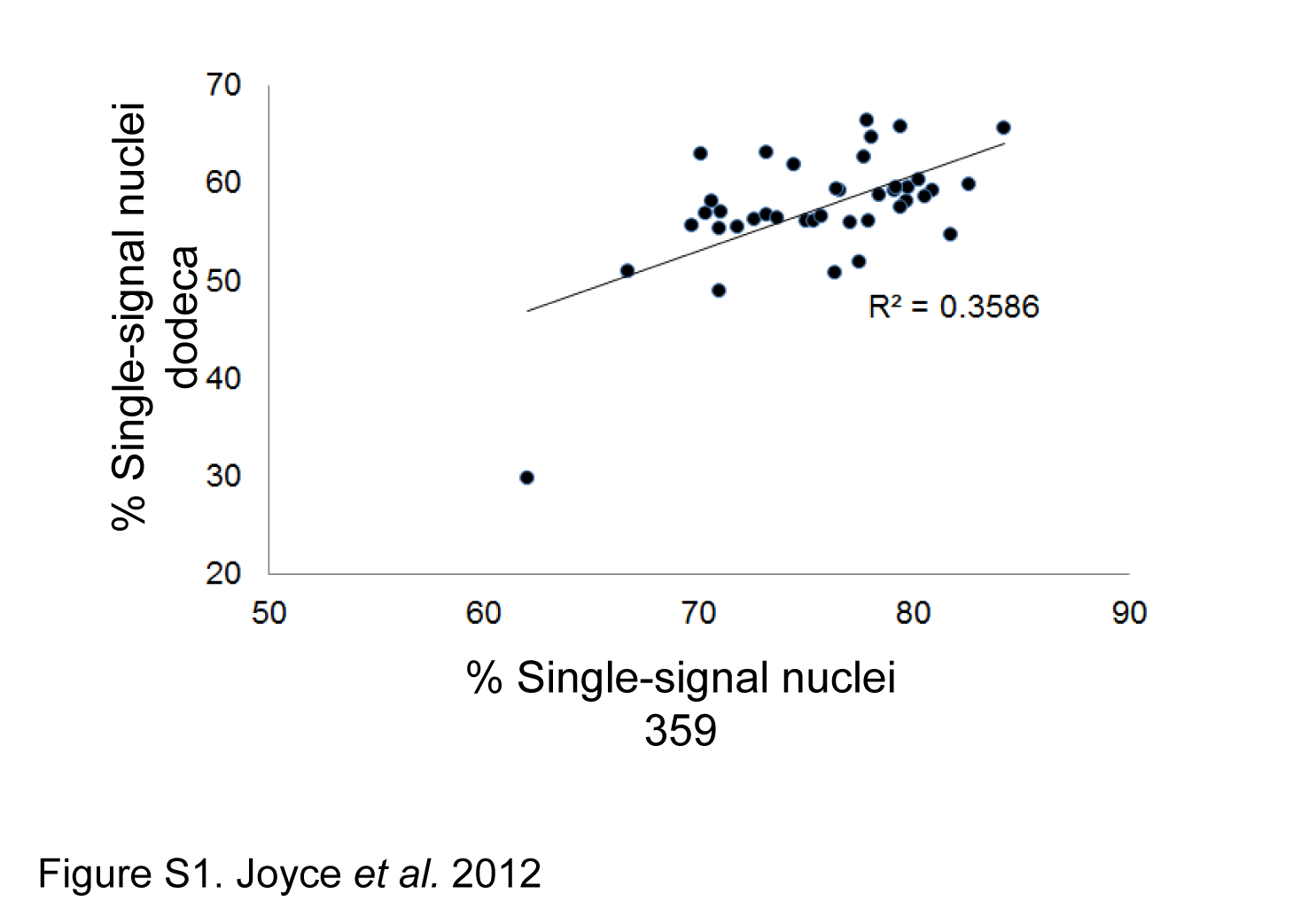

Supplement: Figure S1 — Correlation between 359 and dodeca pairing following RNAi knockdown of pairing promoters. The results from RNAi of all 40 pairing promoters are plotted. X-axis is the percentage of single-signal nuclei at 359. The Y-axis is the percentage of single-signal nuclei at dodeca. The coefficient of determination R2 = 0.3586 represents significant fit of the data to a linear regression, suggesting that pairing levels between the two chromosomal regions are correlative. A minimum number of 250 nuclei were scored for each dsRNA. (TIF) [file pgen.1002667.s001.tif]

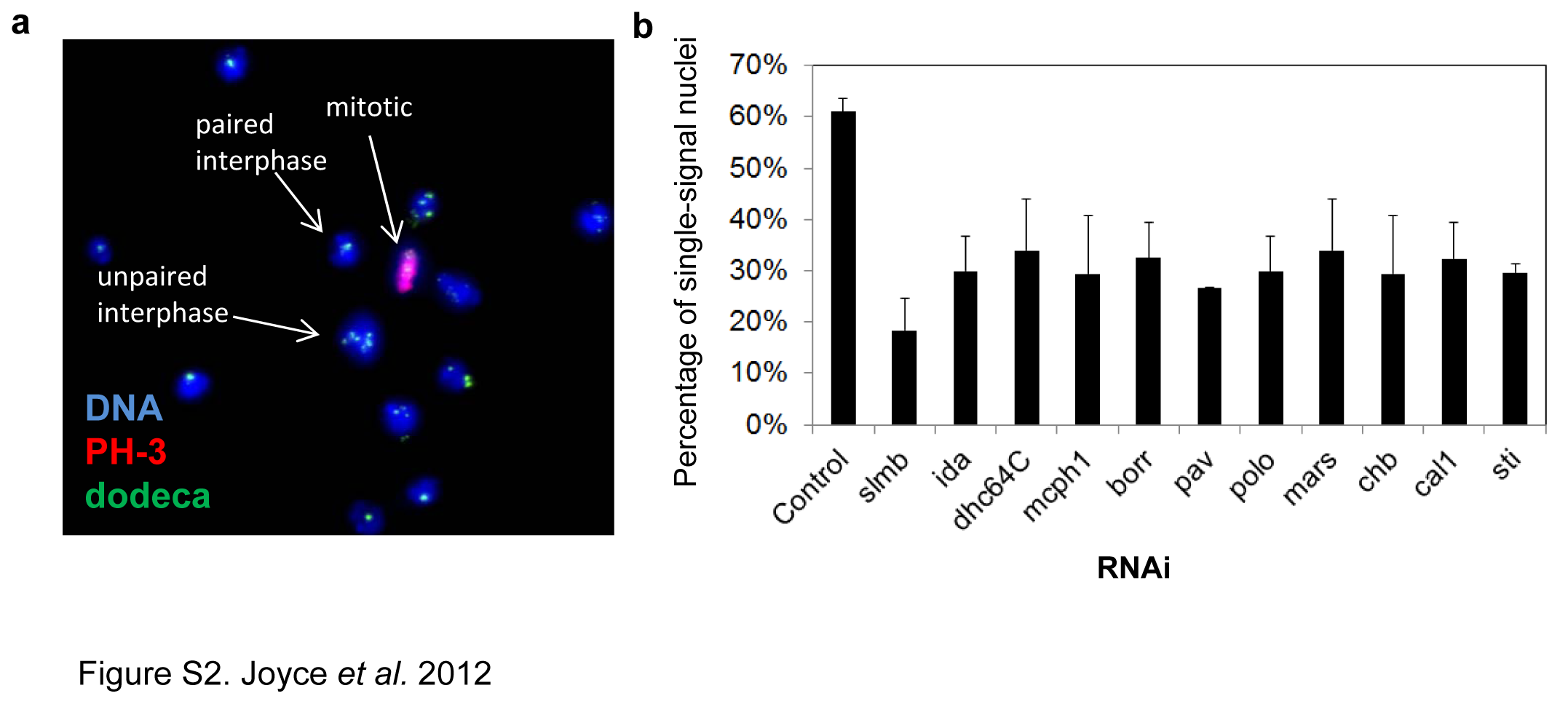

Supplement: Figure S2 — Pairing promoters are important for pairing in interphase nuclei. a, Representative image showing both interphase (PH-3-minus) and mitotic (PH-3-positive) nuclei with dodeca FISH. b, Following depletion of 11 representative pairing promoters, the percentage of single-signal nuclei was significantly decreased compared to control (P<0.05). Error bars denote SD. A minimum number of 100 nuclei were scored for each dsRNA. (TIF) [file pgen.1002667.s002.tif]

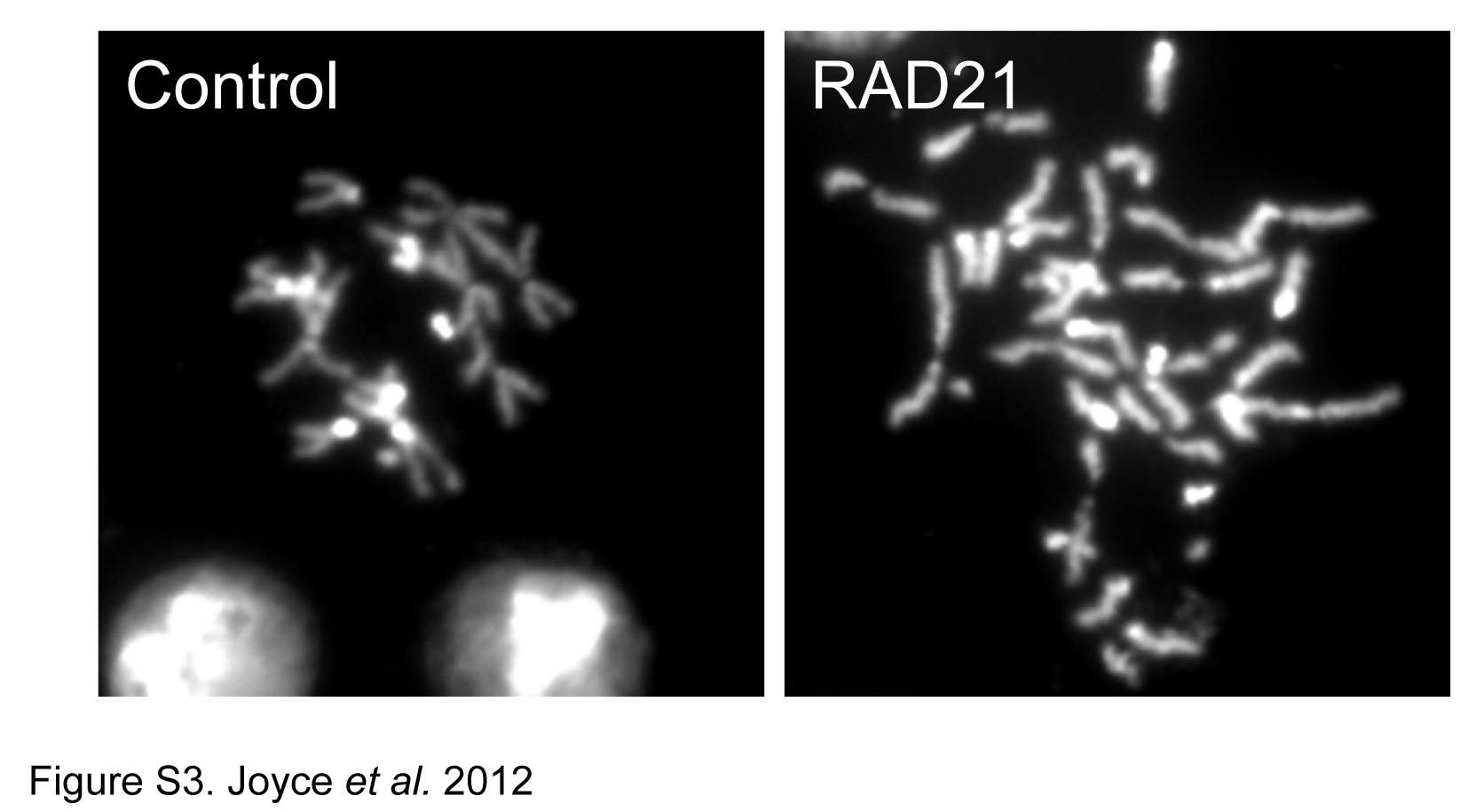

Supplement: Figure S3 — RAD21 depletion leads to premature sister chromatid separation during mitosis. Chromosomes from control metaphase cell with paired sister chromatids and from a RAD21 RNAi metaphase cell clearly showing separated sister chromatids. (TIF) [file pgen.1002667.s003.tif]

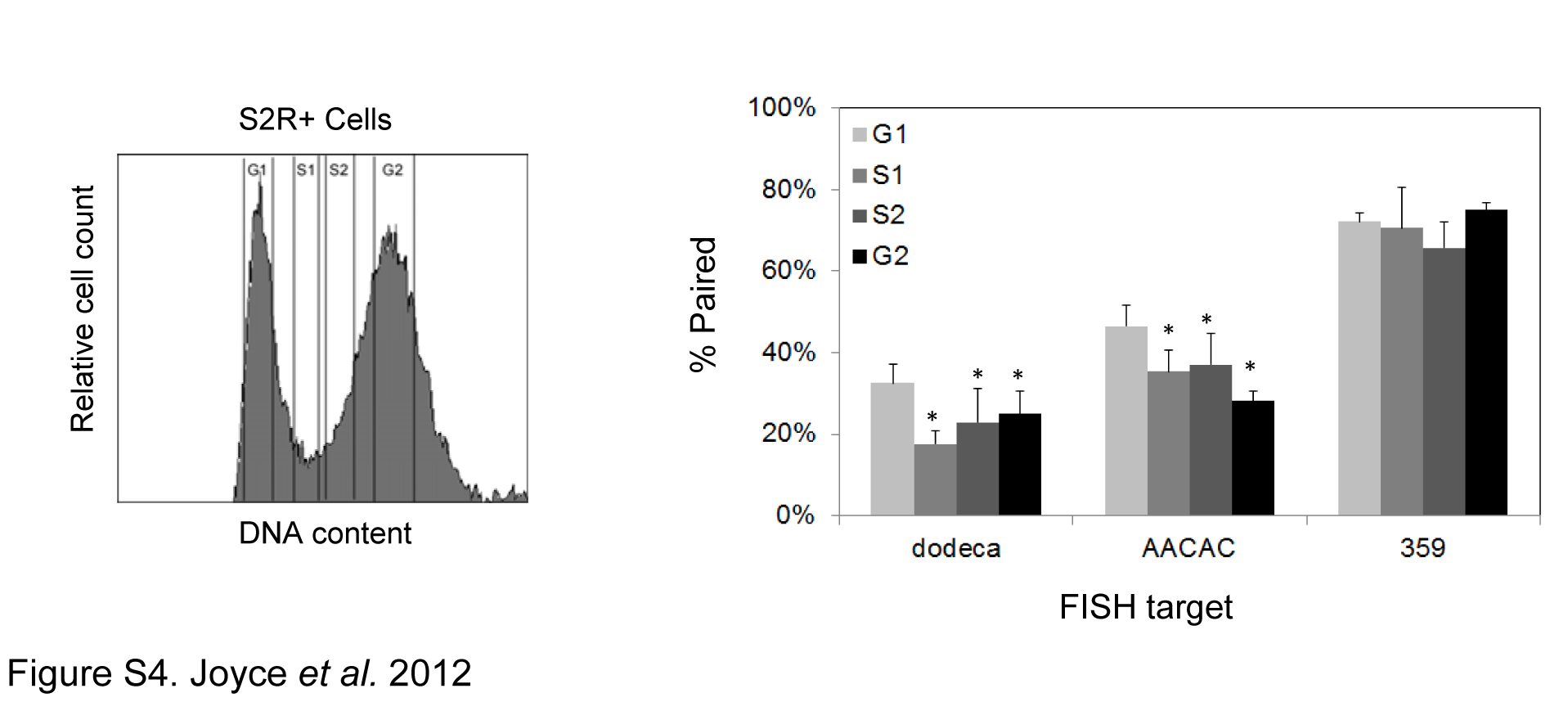

Supplement: Figure S4 — Heterochromatic pairing through the cell cycle in S2R+ cells. FACS plot of S2R+ cells with four gates for G1, early S (S1), late S (S2), and G2 phases of the cell cycle. The frequency ± SD of paired nuclei when targeting 359, AACAC, and dodeca in the G1, S1, S2, and G2 subpopulations. Asterisks denote a significant reduction in paired nuclei at each locus compared to that of G1 cells (P<0.05). A minimum number of 100 nuclei were scored for each subpopulation. (TIF) [file pgen.1002667.s004.tif]
